# A Reinforcement Learning-Based Bi-Population Nutcracker Optimizer for Global Optimization

**DOI:** 10.3390/biomimetics9100596

**Published:** 2024-10-01

**Authors:** Yu Li, Yan Zhang

**Affiliations:** 1 School of Aeronautics and Astronautics, Shenzhen Campus of Sun Yat-sen University, Shenzhen 518000, China; hear4u@163.com; 2 School of Electronics and Communication Engineering, Shenzhen Campus of Sun Yat-sen University, Shenzhen 518000, China

**Keywords:** nutcracker optimizer algorithm, reinforcement learning, bi-population, optimization

## Abstract

The nutcracker optimizer algorithm (NOA) is a metaheuristic method proposed in recent years. This algorithm simulates the behavior of nutcrackers searching and storing food in nature to solve the optimization problem. However, the traditional NOA struggles to balance global exploration and local exploitation effectively, making it prone to getting trapped in local optima when solving complex problems. To address these shortcomings, this study proposes a reinforcement learning-based bi-population nutcracker optimizer algorithm called RLNOA. In the RLNOA, a bi-population mechanism is introduced to better balance global and local optimization capabilities. At the beginning of each iteration, the raw population is divided into an exploration sub-population and an exploitation sub-population based on the fitness value of each individual. The exploration sub-population is composed of individuals with poor fitness values. An improved foraging strategy based on random opposition-based learning is designed as the update method for the exploration sub-population to enhance diversity. Meanwhile, Q-learning serves as an adaptive selector for exploitation strategies, enabling optimal adjustment of the exploitation sub-population’s behavior across various problems. The performance of the RLNOA is evaluated using the CEC-2014, CEC-2017, and CEC-2020 benchmark function sets, and it is compared against nine state-of-the-art metaheuristic algorithms. Experimental results demonstrate the superior performance of the proposed algorithm.

## 1. Introduction

Metaheuristic algorithms, as a class of optimization techniques, are specifically engineered to address complex optimization problems that are challenging or infeasible to solve using traditional methods [1,2]. These algorithms are inspired by natural phenomena, biological processes, physical systems, or social behaviors, offering flexible frameworks for identifying solutions within a reasonable time frame [3,4]. Their advantages, such as simple structure, ease of implementation, and robustness to initial values, have led to their widespread application across various fields, such as power system optimization [5,6], industrial design [7,8], path planning [9,10], and parameter optimization [11,12].

Metaheuristic algorithms rely on two fundamental concepts: exploration and exploitation [13]. These concepts are essential for effectively navigating the search space to identify optimal or near-optimal solutions for complex optimization problems [14]. Exploration involves the algorithm’s capability to explore the broader search space and uncover new regions that may harbor promising solutions [15]. Exploration aims to avoid local optima and ensure a broad investigation of various areas within the search space. In contrast, exploitation involves focusing the search on specific regions that have previously shown promise, with the goal of refining solutions and converging toward the optimal solution by thoroughly searching near high-quality solutions [16]. A significant challenge in the design of metaheuristic algorithms is achieving an appropriate balance between exploration and exploitation [17].

In recent years, numerous metaheuristic algorithms have been proposed, including the grey wolf optimizer (GWO) [18], snake optimizer (SO) [19], white shark optimizer (WSO) [20,21], reptile search algorithm (RSA) [22], crested porcupine optimizer (CPO) [23], and nutcracker optimizer algorithm (NOA) [24]. Among these, the NOA mimics the search, caching, and recovery behaviors of nutcrackers, incorporating two exploration strategies and two exploitation strategies that enhance its fast convergence and robust search capabilities. However, in NOA, the transition between search strategies is governed by random numbers. When applied to complex problems, the NOA encounters limitations, such as an inadequate balance between exploration and exploitation and a propensity to become trapped in local optima.

Several techniques have been adopted to improve the performance of metaheuristic algorithms. The local search method focuses on exploring the neighborhood of a solution to find improvements, enabling the metaheuristic algorithms to escape local optima and continue the search for a global optimum. The authors of ref. [25] proposed a novel local search strategy to improve the particle swarm optimization (PSO) algorithm. After optimizing the population in each iteration, a local search strategy is introduced to enhance the present individuals in the population to accelerate the searching process and prevent becoming trapped in local optima. To improve the population diversity and convergence ability, ref. [26] proposed a variant of GWO with the fusion of a stochastic local search technique, evolutionary operators, and a memory mechanism. The stochastic local search can check the neighborhood of each individual to promote GWO’s exploitation performance. The authors of ref. [27] presented a local search and chaos mapping-based binary group teaching optimization algorithm called BGTOALC. Local search was introduced to increase exploitation. The authors of ref. [28] proposed an oppositional chaotic local search strategy to improve the aquila optimizer. Local search techniques play a critical role in refining solutions within metaheuristic algorithms. However, their embedding may cause the optimizer to perform more exploitation operations during the iterative process. This could exacerbate the imbalance between exploitation and exploration in metaheuristic algorithms.

An elite mechanism is a technique used to preserve the best-performing individuals across iterations for metaheuristic algorithms. The authors of ref. [29] proposed an elite symbiotic organism search algorithm called Elite-SOS. The global convergence ability was enhanced by using the evolutionary information of elite individuals. The authors of ref. [30] built an elite gene pool to guide the reproduction operator and acquire superior offspring. To improve the optimization performance of PSO, ref. [31] built three types of elite archives to save elite individuals with different ranks. Elite individuals could be retained directly during the iteration process, which can make full use of the whole population’s information. The authors of ref. [32] introduced an elite-guided hierarchical mutation strategy to improve the performance of the differential evolution (DE) algorithm. Elite individuals were scheduled for a local search, and the remaining individuals performed a global search guided by the former. The elite mechanism speeds up convergence by ensuring the information of the best solutions persist across generations. However, by focusing on the best solutions, the algorithm might overly emphasize exploitation at the cost of exploration. This imbalance can result in the algorithm getting trapped in local optima.

Incorporating supervised learning into metaheuristic algorithms is an emerging area of research that uses training knowledge to assist in the acquisition of optimal solutions in the iterative process. The authors of ref. [33] proposed a kernelized autoencoder that can learn from past search experiences to speed up the optimization process. The authors of ref. [34] presented autoencoding to predict the moving of the optimal solutions. To solve the problems of parameter setting and strategy selection, ref. [35] proposed an adaptive distributed DE algorithm. The individual and population parameters were updated adaptively based on the best solutions and historically successful experience. The authors of ref. [36] introduced a learning-aided evolutionary optimization framework that learns knowledge from the historical optimization process by using artificial neural networks. The learned knowledge can help metaheuristic algorithms to better approach the global optimum. While supervised learning can guide the search process more effectively, the training phase requires additional computational resources and is not suitable for time-constrained problems. In addition, the generalization of supervised learning also limits the application scenarios of this kind of strategy.

Reinforcement learning (RL) is a subfield of machine learning in which an agent learns to make decisions by taking actions within an environment to maximize cumulative rewards [37,38]. Due to its strong environmental interaction capabilities, RL has been increasingly employed by researchers to guide the selection of search strategies in metaheuristic algorithms. The authors of ref. [39] introduced an inverse reinforcement learning-based moth-flame optimization algorithm, IRLMFO, to solve large-scale optimization problems. RL was utilized to select effective search strategies based on historical data from the strategy pool established by IRLMFO. To overcome the drawbacks of getting trapped in local optima easily, ref. [40] presented a reinforcement learning-based RSA known as RLNSA, where RL managed the switching between exploration and exploitation strategies. Additionally, refs. [41,42] applied RL to address mutation strategy selection within the evolutionary process of differential evolution algorithms. The authors of ref. [43] embedded RL in the teaching–learning-based optimization algorithm (RLTLBO) to solve optimization problems. The authors of ref. [44] proposed a reinforcement learning-based memetic particle swarm optimization algorithm called RLMPSO. The selection of five search operations is controlled by the RL algorithm. The authors of ref. [45] designed a reinforcement learning-based comprehensive learning grey wolf optimizer (RLCGWO) to adaptively adjust strategies. Although the introduction of RL can enable metaheuristic algorithms to adaptively select exploration and exploitation strategies, it does not always effectively enhance algorithm performance. Typically, exploration is enhanced through methods such as large step sizes, random perturbations, or probabilistic jumps, which enable the algorithm to search beyond the current solutions [46]. Consequently, in RL-based metaheuristic algorithms, exploration strategies often receive rewards mainly during the early optimization stages, leading RL to favor exploitation strategies as optimization progresses. This tendency can cause existing RL-based metaheuristic algorithms to struggle in escaping local optima, as exploration strategies are less frequently selected.

To overcome the aforementioned problems, this paper introduces an RL-based bi-population NOA called RLNOA. The RLNOA introduces a bi-population mechanism to better balance exploration and exploitation in the optimization process. At the beginning of each iteration, the population is divided into the exploration sub-populations and the exploitation sub-populations. Individuals with poor fitness in the raw population form the exploration sub-population. A random opposition-based learning (ROBL)-based foraging method is proposed as the update strategy for the exploration sub-population to avoid local optima. The remaining outstanding individuals of the raw population formed the exploitation sub-populations, which use Q-learning within RL to adaptively select between the NOA’s two exploitation strategies (storage and recovery) to accelerate convergence and improve generalization. The division of these sub-populations is based on fitness ranking and optimization progress. Experimental results show that the RLNOA achieves superior optimization performance compared to current state-of-the-art algorithms. The primary contributions of this paper are as follows:

- An RL-based bi-population nutcracker optimizer algorithm (RLNOA) is developed to solve complex optimization problems;
- The foraging strategy of the NOA is enhanced using ROBL, improving its ability to search for feasible solutions;
- Q-learning is utilized to control the selection of the most appropriate exploitation strategy for each iteration, dynamically improving the refinement of the optimal solution.

The remainder of this paper is organized as follows. Section 2 describes the NOA and RL methods. Section 3 explains the detailed implementation of the proposed RLNOA. The comparison experiments are finished in Section 4. Section 5 summarizes the conclusions.

## 2. Preliminaries

### 2.1. Nutcracker Optimization Algorithm

The NOA is a metaheuristic algorithm inspired by the natural behavior of nutcrackers [24]. It solves optimization problems by simulating the nutcracker’s behavior in collecting, storing, and searching for food. The optimization process in the NOA is carried out through four strategies: foraging, storage, cache search, and recovery. Table A1 summarizes the nomenclature of this study.

#### 2.1.1. Foraging and Storage Strategies

During the foraging phase, individuals start searching for potential food sources within the search space. This behavior is mathematically modeled as follows:

(1) $$x_{i,j}^{t+1\left(FSnew_{1}\right)}=\left\{\begin{array}{l} x_{i,j}^{t}\;,\;if\;rand_{1}<rand_{2}\\ {x_{i,j}^{t}}^{\prime }\;,\;otherwise \end{array}\right.$$

(2) $${x_{i,j}^{t}}^{\prime }=\left\{\begin{array}{ll} x_{m,j}^{t}+\varepsilon \cdot \left(x_{A,j}^{t}-x_{B,j}^{t}\right)+\mu \cdot \left(r_{1}^{2}\cdot U_{j}-L_{j}\right) & \;,\;if\;t<T_{\max}/2\\ x_{C,j}^{t}+\mu \cdot \left(x_{A,j}^{t}-x_{B,j}^{t}\right)+\mu \cdot \left(r_{2}<\delta \right)\cdot \left(r_{3}^{2}\cdot U_{j}-L_{j}\right) & \;,\;otherwise \end{array}\right.$$

where $x_{i,j}^{t+1\left(FSnew_{1}\right)}$ is the new position of the *i*th individual generated in the foraging phase; $x_{i,j}^{t}$ is the *j*th dimension of the *i*th individual in the iteration t; $x_{m,j}^{t}$ is the mean position of the *j*th dimensions for the current population in the iteration t; $T_{\max}$ indicates the maximum generations; $L_{j}$ and $U_{j}$ are the lower and upper bounds of the optimization problem in the *j*th dimension; *A*, *B*, and *C* are three different integers randomly selected in the range of [0, *NP*]; *NP* is the population size; ε is a parameter generated by the levy flight; the values of $rand_{1}$, $rand_{2}$, $r_{1}$, $r_{2}$, and $r_{3}$ are random numbers selected within the range [0, 1]; δ is a control parameter; and μ is a parameter chosen among $\tau_{1}$ (chosen randomly between zero and one), $\tau_{2}$ (the normal distribution), and $\tau_{3}$ (levy flight), as follows:

(3) $$\mu =\left\{\begin{array}{l} \tau_{1}\;,\;if\;rand_{1}<rand_{2}\\ \tau_{2}\;,\;if\;rand_{2}<rand_{3}\\ \tau_{3}\;,\;if\;rand_{1}<rand_{3} \end{array}\right.$$

where $rand_{1}$, $rand_{2}$, and $rand_{3}$ are random numbers selected within the range [0, 1].

At the storage phase, individuals store foods as follows:

(4) $$x_{i}^{t+1\left(FSnew_{2}\right)}=\left\{\begin{array}{ll} x_{i}^{t}+\mu \cdot \left(x_{best}^{t}-x_{i}^{t}\right)\cdot \left|\lambda \right|+r_{1}\cdot \left(x_{A}^{t}-x_{B}^{t}\right) & ,\;if\;rand_{1}<rand_{2}\\ x_{best}^{t}+\mu \cdot \left(x_{A}^{t}-x_{B}^{t}\right) & \;,\;if\;rand_{1}<rand_{3}\\ x_{best}^{t}\cdot \xi & \;,\;otherwise \end{array}\right.$$

where $x_{i}^{t+1\left(FSnew_{2}\right)}$ is the new position of the *i*th individual generated in the storage phase; λ is a parameter generated by the levy flight; and $r_{1}$, $rand_{1}$, $rand_{2}$, and $rand_{3}$ are random numbers selected within the range [0, 1]. ξ is a parameter that linearly decreased from 1 to 0 during the optimization process.

The exchange between the foraging and storage strategies is used to balance exploration and exploitation phases as follows:

(5) $$x_{i}^{t+1\left(FS\right)}=\left\{\begin{array}{l} x_{i}^{t+1\left(FSnew_{1}\right)}\;,\;if\;rand_{1}<P_{a1}\\ x_{i}^{t+1\left(FSnew_{2}\right)}\;,\;otherwise \end{array}\right.$$

where $rand_{1}$ is random numbers selected within the range [0, 1] and $P_{a1}$ is a parameter that linearly decreased from 1 to 0 during the optimization process.

#### 2.1.2. Cache Search and Recovery Strategies

At the cache search phase, individuals locate their caches through two reference points:

(6) $$RP_{i,1}^{t}=\left\{\begin{array}{l} x_{i}^{t}+\alpha \cdot \cos\left(\theta \right)\cdot \left(x_{A}^{t}-x_{B}^{t}\right)+\beta \cdot RP_{i,randi_{1↔2}}^{t}\;,\;if\;\theta =\frac{\pi }{2}\\ x_{i}^{t}+\alpha \cdot \cos\left(\theta \right)\cdot \left(x_{A}^{t}-x_{B}^{t}\right)\;,\;otherwise \end{array}\right.$$

(7) $$RP_{i,2}^{t}=\left\{\begin{array}{l} x_{i}^{t}+\left(\alpha \cdot \cos\left(\theta \right)\left(\left(U-L\right)\cdot r_{1}+L\right)+\alpha \cdot RP_{i,randi_{1↔2}}^{t}\right)\cdot \xi \;,\;if\;\theta =\frac{\pi }{2}\\ x_{i}^{t}+\alpha \cdot \cos\left(\theta \right)\cdot \left(\left(U-L\right)\cdot r_{1}+L\right)\cdot \xi \;,\;otherwise \end{array}\right.$$

and

(8) $$\xi =\left\{\begin{array}{l} 1\;,\;if\;rand_{1}<P_{rp}\\ 0\;,\;otherwise \end{array}\right.$$

where θ is a parameter chosen in the range of [0, π]; $randi_{1↔2}$ is an integer chosen randomly between zero and one; $r_{1}$, $rand_{1}$ are random numbers selected within the range [0, 1]; $P_{rp}$ is a global exploration threshold; and β is a convergence parameter and can be acquired as follows:

(9) $$\beta =\left\{\begin{array}{ll} {\left(1-\frac{t}{T_{\max}}\right)}^{2\frac{t}{T_{\max}}} & ,\;if\;rand_{1}<rand_{2}\\ {\left(\frac{t}{T_{\max}}\right)}^{\frac{2}{t}} & \;,\;otherwise \end{array}\right.$$

where $rand_{1}$ and $rand_{2}$ are random numbers selected within the range [0, 1]. The new position of the individual during the cache search phase can be acquired as follows:

(10) $$x_{i,j}^{t+1\left(CRnew_{1}\right)}=\left\{\begin{array}{l} x_{i,j,1}^{t}\;,\;if\;rand_{1}<rand_{2}\\ x_{i,j,2}^{t}\;,\;otherwise \end{array}\right.$$

(11) $$x_{i,j,1}^{t+1}=\left\{\begin{array}{ll} x_{i,j}^{t} & \;,\;if\;rand_{1}<rand_{2}\\ x_{i,j}^{t}+r_{1}\cdot \left(x_{best,j}^{t}-x_{i,j}^{t}\right)+r_{2}\cdot \left(RP_{i,j,1}^{t}-x_{C,j}^{t}\right) & \;,\;otherwise \end{array}\right.$$

(12) $$x_{i,j,2}^{t+1}=\left\{\begin{array}{ll} x_{i,j}^{t} & \;,\;if\;rand_{1}<rand_{2}\\ x_{i,j}^{t}+r_{1}\cdot \left(x_{best,j}^{t}-x_{i,j}^{t}\right)+r_{2}\cdot \left(RP_{i,j,2}^{t}-x_{C,j}^{t}\right) & \;,\;otherwise \end{array}\right.$$

where $RP_{i,j,1}^{t}$ and $RP_{i,j,2}^{t}$ are the *j*th position of $RP_{i,1}^{t}$ and $RP_{i,2}^{t}$, respectively; $rand_{1}$, $rand_{2}$, $r_{1}$, and $r_{2}$ are random numbers selected within the range [0, 1]; and *C* is the index of a solution selected randomly from the population.

During the recovery phase, nutcrackers find the hidden caches and retrieve the buried pine seeds. The new position of a nutcracker is obtained using the following equation:

(13) $$x_{i,j}^{t+1\left(CRnew_{2}\right)}=\left\{\begin{array}{l} RP_{i,1}^{t}\;,\;if\;f\left(RP_{i,1}^{t}\right)<f\left(RP_{i,2}^{t}\right)\;and\;f\left(RP_{i,1}^{t}\right)<f\left(x_{i}^{t}\right)\;\\ RP_{i,1}^{t}\;,\;if\;f\left(RP_{i,2}^{t}\right)<f\left(RP_{i,1}^{t}\right)\;and\;f\left(RP_{i,2}^{t}\right)<f\left(x_{i}^{t}\right)\\ x_{i}^{t}\;,\;otherwise\; \end{array}\right.$$

Finally, the exchange between the cache search and recovery strategies is applied according to the following formula:

(14) $$x_{i}^{t+1\left(CR\right)}=\left\{\begin{array}{l} x_{i}^{t+1\left(CRnew_{1}\right)}\;,\;if\;rand_{1}>P_{a2}\\ x_{i}^{t+1\left(CRnew_{2}\right)}\;,\;otherwise \end{array}\right.$$

where $rand_{1}$ is random numbers selected within the range [0, 1] and $P_{a2}$ represents a probability value.

### 2.2. Reinforcement Learning

RL has been applied across various domains due to its effectiveness in problem-solving [47,48]. In RL, the agent interacts with the environment to learn how to perform optimal actions. As the representative method of RL, Q-learning defines the Q-table to control an agent’s actions. The Q-table is an *m* × *n* matrix, where m represents the number of states and n represents the number of actions available to the agent. By making a decision in the current state based on the Q-table values, the agent ultimately maximizes its reward. The Q-table is dynamically updated as follows:

(15) $$Q\left(s_{t+1},a_{t+1}\right)=Q\left(s_{t},a_{t}\right)+\alpha \left[R_{t+1}+\gamma \underset{a}{\max}Q\left(s_{t+1},a\right)-Q\left(s_{t},a_{t}\right)\right]$$

where $s_{t}$ and $s_{t+1}$ represent the current and next states, respectively; $a_{t}$ and $a_{t+1}$ represent the current and next actions, respectively; $R_{t+1}$ is the reward acquired after performing action $a_{t}$; α is the learning rate; γ is the discount factor; and $Q\left(\cdot \right)$ represents the corresponding value in the Q-table.

## 3. The Development of the Proposed Algorithm

### 3.1. Overview

The traditional NOA is limited to specific problems and is prone to getting trapped in local optima. To overcome these limitations, this paper introduces a hybrid strategy called the RLNOA. In each iteration of the RLNOA, the population is segmented into two groups based on fitness ranking: the exploration sub-population and the exploitation sub-population. The exploration sub-population consists of individuals with poor fitness in the current population. A ROBL-based foraging strategy is employed as the update strategy for individuals in the exploration sub-population, with a sine-based perturbation introduced to adjust the size of the exploration sub-population to ensure convergence. The remaining individuals form the exploitation sub-population, which implements two types of exploitation strategies: storage and recovery. The selection of the exploitation strategy is governed by Q-learning.

**Figure 1:**
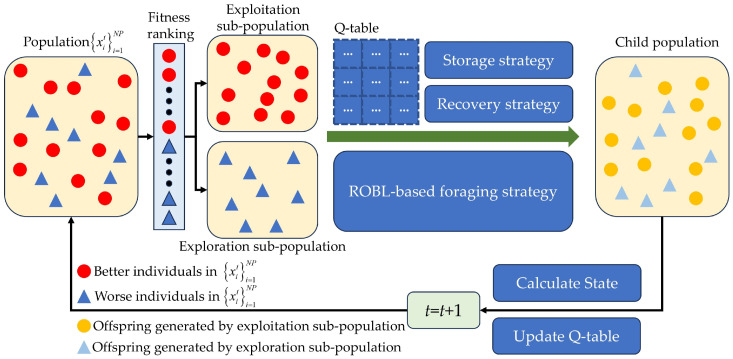
Illustration of the RLNOA.

Furthermore, the RLNOA utilizes a single Q-table to map individual states to actions. States in the RLNOA are encoded by relative changes in fitness value and local diversity, while actions correspond to exploitation strategies. The exploitation sub-population updates based on the strategy selected by each individual, generating offspring. Rewards are assigned based on the selection process outcomes, and the Q-table is subsequently updated for the next generation. Figure 1 illustrates the flowchart of the RLNOA, with the main steps detailed in Algorithm 1.
**Algorithm 1:** The pseudocode of the RLNOA$\mathrm{Input}:\;\mathrm{Population}\;\mathrm{size}\;\mathrm{NP},\;\mathrm{Maximum}\;\mathrm{number}\;\mathrm{of}\;\mathrm{generations}\;T_{\max}$ Learning rate λ Discount factor γ, $,\;P_{rp}$, δ $\mathrm{Output}:\;\mathrm{The}\;\mathrm{best}\;\mathrm{solution}\;x_{best}$$\;\mathrm{and}\;\mathrm{corresponding}\;\mathrm{fitness}\;\mathrm{value}\;f\left(x_{best}\right)$. Set the initial Q-table: Q(*s*, *a*) = 0 Set *t* = 1 $\mathrm{Initialize}\;\mathrm{population}\;\mathrm{position}:\;x_{i}^{t},i=1,\;2,\;\ldots ,\;NP$ $\mathrm{Calculate}\;\mathrm{the}\;\mathrm{fitness}\;\mathrm{values}\;\mathrm{for}\;\mathrm{each}\;\mathrm{individual}:\;f\left(x_{i}^{t}\right)$ $\mathrm{Calculate}\;\mathrm{the}\;\mathrm{local}\;\mathrm{diversity}\;\mathrm{for}\;\mathrm{each}\;\mathrm{individual}:\;D_{i}^{t}$ $\mathrm{Set}\;D_{i}^{t-1}=D_{i}^{t}$$,\;f\left(x_{i}^{t-1}\right)=f\left(x_{i}^{t}\right)$ **While** *t* < *T*_max_ **do**    Acquire sub-population by Equation (19)    $\mathrm{For}\;\mathrm{each}\;\mathrm{individual}\;x_{i}^{t}$       $\mathrm{If}\;x_{i}^{t}$ belong to exploration sub-population          $\mathrm{Perform}\;\mathrm{ROBL}\text{-}\mathrm{based}\;\mathrm{foraging}\;\mathrm{strategy}\;\mathrm{on}\;x_{i}^{t}$ by Equation (16)       **Else**          Determine the state of the exploitation sub-population by Equations (20) and (21)          Choose the best *a* for the current *s* from Q-table          **Switch** action              **Case 1:** Storage                  $\mathrm{Perform}\;\mathrm{storage}\;\mathrm{strategy}\;\mathrm{on}\;x_{i}^{t}$ by Equation (4)              **Case 2:** Recovery                   $\mathrm{Perform}\;\mathrm{recovery}\;\mathrm{strategy}\;\mathrm{on}\;x_{i}^{t}$ by Equation (13)          **End Switch**          Set the reward by Equation (25)       **End if**       $\mathrm{Calculate}\;\mathrm{the}\;\mathrm{fitness}\;f\left(x_{i}^{t}\right)$$\;\mathrm{of}\;x_{i}^{t}$       $\mathrm{Update}\;\mathrm{the}\;\mathrm{position}\;\mathrm{of}\;x_{i}^{t}$ if its fitness is improved    **End for**    Calculate the relative changes of fitness and local diversity for the population    Update Q-table by the exploitation sub-population    *t* = *t* + 1 **End While** **Return results** **Terminate**

### 3.2. ROBL-Based Foraging Strategy

**Figure 2:**
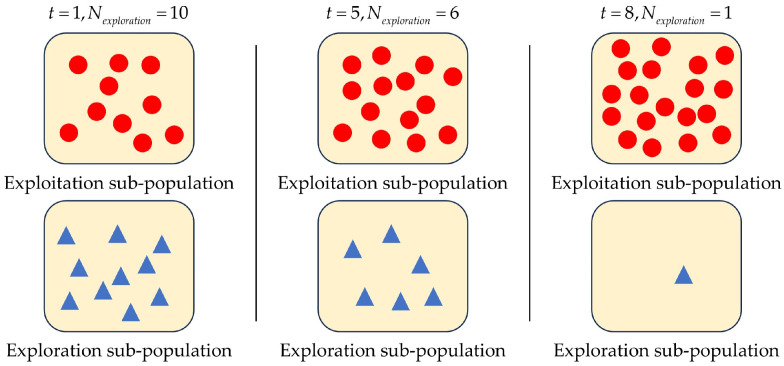
The variation of $N_{exploration}$ when $T_{\max}=10$, NP=20, and ζ=8.

In the RLNOA, based on the ROBL method, an improved foraging strategy is introduced to construct the exploration behavior for the exploration sub-population [18]. The offspring in the exploration sub-population can be generated as follows:

(16) $$x_{i,j}^{t+1\left(new_{1}\right)}=\left\{\begin{array}{l} {x_{i,j}^{t}}^{\prime }\;,\;if\;t<T_{\max}/2\\ {x_{i,j}^{t}}^{\prime \prime }\;,\;otherwise \end{array}\right.$$

(17) $${x_{i,j}^{t}}^{\prime }=\left\{\begin{array}{ll} x_{m,j}^{t}+\varepsilon \cdot \left(x_{A,j}^{t}-x_{B,j}^{t}\right)+\mu \cdot \left(r_{1}^{2}\cdot U_{j}-L_{j}\right) & ,\;if\;rand_{1}<rand_{2}\\ L_{j}+U_{j}-r_{1}\cdot x_{i.j}^{t} & \;,\;otherwise \end{array}\right.$$

(18) $${x_{i,j}^{t}}^{\prime \prime }=\left\{\begin{array}{ll} x_{C,j}^{t}+\mu \cdot \left(x_{A,j}^{t}-x_{B,j}^{t}\right)+\mu \cdot \left(r_{2}<\delta \right)\cdot \left(r_{3}^{2}\cdot U_{j}-L_{j}\right) & ,\;if\;rand_{1}<rand_{2}\\ L_{j}+U_{j}-r_{2}\cdot x_{i.j}^{t} & \;,\;otherwise \end{array}\right.$$

where $x_{i,j}^{t+1\left(new_{1}\right)}$ is the new position of the *i*th individual generated in the foraging phase; $x_{i,j}^{t}$ is the *j*th dimension of the *i*th individual in the iteration t; $x_{m,j}^{t}$ is the mean position of the *j*th dimensions for the current population in the iteration t; $T_{\max}$ indicates the maximum generations; $L_{j}$ and $U_{j}$ are the lower and upper bounds of the optimization problem in the *j*th dimension; *A*, *B*, and *C* are three different integers randomly selected in the range of [0, *NP*]; *NP* is the population size; ε is a parameter generated by the levy flight; $rand_{1}$, $rand_{2}$, $r_{1}$, $r_{2}$, and $r_{3}$ are random numbers selected within the range [0, 1]; δ is a control parameter; and μ is a parameter generated based on Equation (3). The size of the exploration sub-population can be calculated as follows:

(19) $$N_{exploration}=round\left[\frac{Np}{2}{\left(1-\sin\left(\frac{\pi }{2}\sqrt{\frac{t}{T_{\max}}}\right)\right)}^{\zeta }\right]$$

where ζ is the control parameter. At the beginning of each iteration, $N_{exploration}$ individuals with poor fitness values are chosen from the total population to form the exploration sub-population. For example, assuming that $T_{\max}=10$, NP=20 and ζ=8. The variation of $N_{exploration}$ is illustrated in Figure 2. To ensure population diversity and prevent premature convergence, the value of $N_{exploration}$ decreases slowly in the early stages of the optimization process. In the later stages, $N_{exploration}$ decays rapidly, allowing most individuals to focus on local exploitation.

### 3.3. Q-Learning-Based Exploitation Behavior

To ensure dynamic optimization of benefits at different stages for solving the optimization problem, Q-learning is employed as the selector for the exploitation sub-population to control the switch between storage (Equation (4)) and recovery (Equation (13)) strategies. The settings for Q-learning are specified as follows:

#### 3.3.1. State Encoding

**Figure 3:**
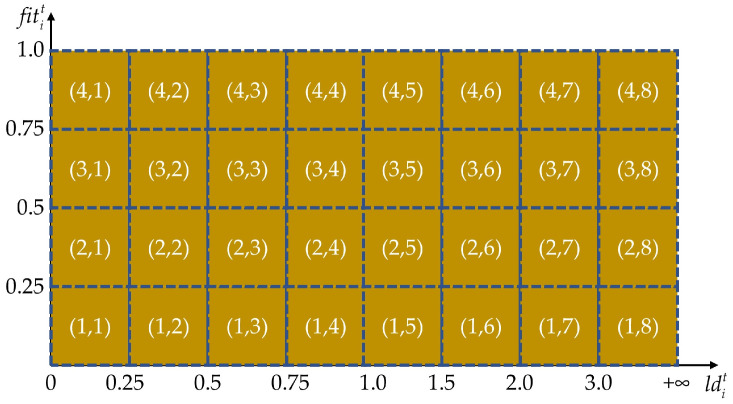
The illustration of states in the exploitation population.

The state of each individual is encoded as the relative changes of local diversity and fitness values, which are defined as follows:

(20) $$ld_{i}^{t}=D_{i}^{t}/D_{i}^{t-1}$$

(21) $$fit_{i}^{t}=f\left(x_{i}^{t}\right)/f\left(x_{i}^{t-1}\right)$$

where $ld_{i}^{t}$ is the relative changes in local diversity; $fit_{i}^{t}$ is the relative changes in fitness value; and $D_{i}^{t}$ represents the local diversity of individual $x_{i}^{t}$ and can be calculated as follows:

(22) $$D_{i}^{t}=\frac{1}{k}\sum_{l=1}^{k}\sqrt{\sum_{j=1}^{d}{\left(x_{l,j}^{t}-{\bar{x}}_{l,j}^{t}\right)}^{2}}$$

(23) $${\bar{x}}_{l,j}^{t}=\frac{1}{k}\sum_{l=1}^{k}x_{l,j}^{t}$$

where ${\left\{x_{l}^{t}\right\}}_{l=1}^{k}$ is the neighborhood set of $x_{i}^{t}$; d is the dimension of the search space; and k is the number of near neighbors. As shown in Figure 3, the exploitation population consists of 32 states in total. Among these, the dimension of ld is divided into eight states: [0, 0.25), [0.25, 0.5), [0.5, 0.75), [0.75, 1.0), [1, 1.5), [1.5, 2), [2, 3), and [3, +∞). Moreover, the dimension of $fit^{t}$ is divided into four states: [0, 0.25), [0.25, 0.5), [0.5, 0.75), and [0.75, 1.0].

#### 3.3.2. Action Options

The action of each individual is encoded as the selection of exploitation strategies. The probability of selecting each action is computed using the SoftMax function:

(24) $$\pi_{q}\left(s_{t},a_{j}\right)=\frac{\exp\left(Q_{t}\left(s_{t},a_{j}\right)\right)}{\sum_{j=1}^{n}\exp\left(Q_{t}\left(s_{t},a_{j}\right)\right)}$$

where $Q_{t}\left(s_{t},a_{j}\right)$ is the value in the Q-table in the iteration t; $a_{j}$ is the *j*th action; and n is the total number of actions. Each individual sample has an optimization strategy based on the probability of each action.

#### 3.3.3. Reward Options

The reward is determined based on the selection results of each generation, reflecting the performance of the current optimization strategy. If the fitness value of the new position $x_{i}^{t+1}$ is better than the old position $x_{i}^{t}$, the individual is rewarded with 1. Otherwise, the individual is punished with −1. The reward settings are defined as follows:

(25) $$R=\left\{\begin{array}{ll} 1 & \;,\;if\;f\left(x_{i}^{t+1}\right)<f\left(x_{i}^{t}\right)\\ -1 & \;,\;otherwise \end{array}\right.$$

Based on the above settings, the Q-table can be updated by Equation (15). The pseudocode of the RLNOA is shown in Algorithm 1.

### 3.4. Time Complexity

As can be seen from the pseudocode of the RLNOA in Algorithm 1, the proposed algorithm mainly consists of the following parts.

(1) Initializing the population and updating the fitness values and local diversity for each individual, with a time complexity of $O\left(NP\times d\right)$.

(2) Acquiring the sub-populations, with a time complexity of $O\left(T_{\max}\times NP\right)$.

(3) Updating the position for the exploration sub-population and exploitation sub-population, with a time complexity of $O\left(T_{\max}\times NP\times d\right)$.

(4) Calculating the relative changes in fitness and local diversity for the populations, with a time complexity of $O\left(T_{\max}\times NP\times d\right)$.

(5) Updating the Q-table using the exploitation sub-population, with a time complexity of $O\left(T_{\max}\times \left(NP-N_{exploration}\right)\times d\right)$.

Therefore, the maximum computing complexity of the RLNOA is $O\left(T_{\max}\times NP\times d\right)$, which is the same as that of the NOA.

## 4. Experimental Results

In this section, we perform a series of experiments on publicly available benchmark problems to assess the effectiveness of the RLNOA. The results are compared and analyzed against other state-of-the-art methods that have shown promising performances in the literature.

### 4.1. Test Conditions

The performance of the proposed RLNOA was tested on three global optimization test suites, including CEC-2014, CEC-2017, and CEC-2020 [49]. These test suites consist of unimodal, multimodal, hybrid, and composition functions, each with only one global optimum. The proposed RLNOA was compared with the NOA [24], SO [19], RSA [22], crested porcupine optimizer (CPO) [23], GWO [10], PSO [3], RLTLBO [43], RLMPSO [44], and RLCGWO [45]. The PSO and GWO are classical algorithms, while the NOA, SO, RSA, and CPO are well-known and recently proposed algorithms. The RLTLBO, RLMPSO, and RLCGWO are RL-based and recently proposed algorithms.

The common parameters for the experimental algorithms are presented in Table 1. The maximum number of iterations is set to 1000. To assess the experimental results, several performance metrics are used, including the average (Ave) and standard deviation (Std) of fitness values from 30 independent runs, and a ranking metric to assess the order of each method according to its average fitness value. Additionally, to highlight the significant differences between the RLNOA’s results and those of competing algorithms, convergence curves and box plots are utilized. The experiments were implemented in MATLAB R2024a on a device with Intel(R) Core(TM) i7-14700KF CPU @ 3.40 GHz and 64 GB RAM.

### 4.2. Comparison over CEC-2014

We performed optimization experiments on the CEC-2014 test suite to verify the effectiveness of the proposed RLNOA. The CEC-2014 test suite is a diverse collection of 30 test functions, including three unimodal functions, 13 multimodal functions, six hybrid functions, and eight composite functions. Each category is designed to test different aspects of an optimization algorithm’s capability, such as its ability to handle multiple local optima, locate the global optimum, and efficiently explore and exploit the search space. These functions are characterized by various levels of difficulty, dimensionality, and complexity, making them comprehensive tools for assessing the robustness, efficiency, and accuracy of optimization algorithms.

Table 2 shows the experimental results of various algorithms applied to the CEC-2014 test suite. As shown in the table, except for F3 and F30, the proposed RLNOA ranks first among all comparative algorithms in the remaining functions. Additionally, the second-to-last row in Table 2 confirms that the RLNOA achieves the highest average ranking, with a value of 1.0345. The second highest is the NOA, with a value of 2.1724, while the SO algorithm performs the worst.

**Figure 4:**
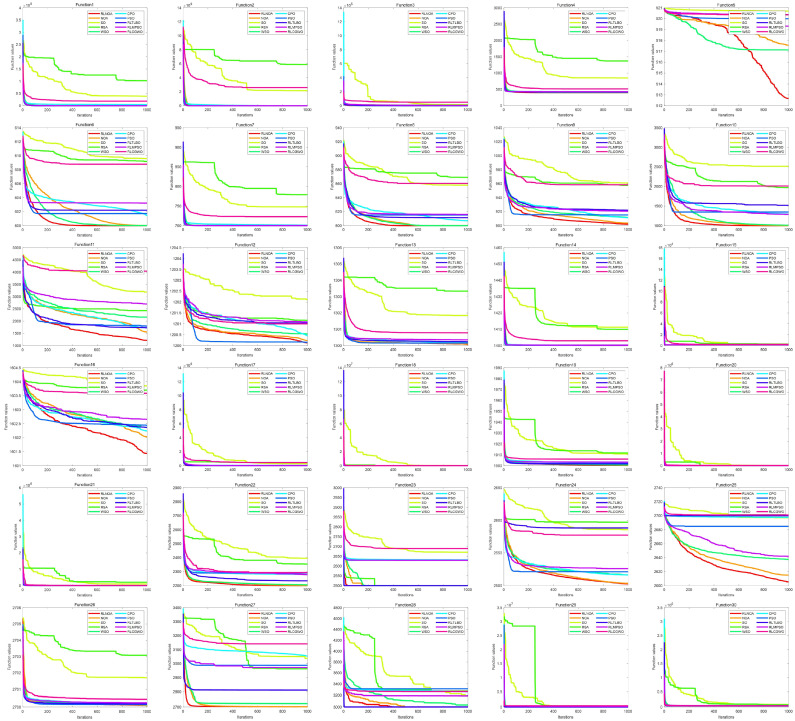
The convergence curves of different algorithms applied to the CEC-2014 test suite.

**Figure 5:**
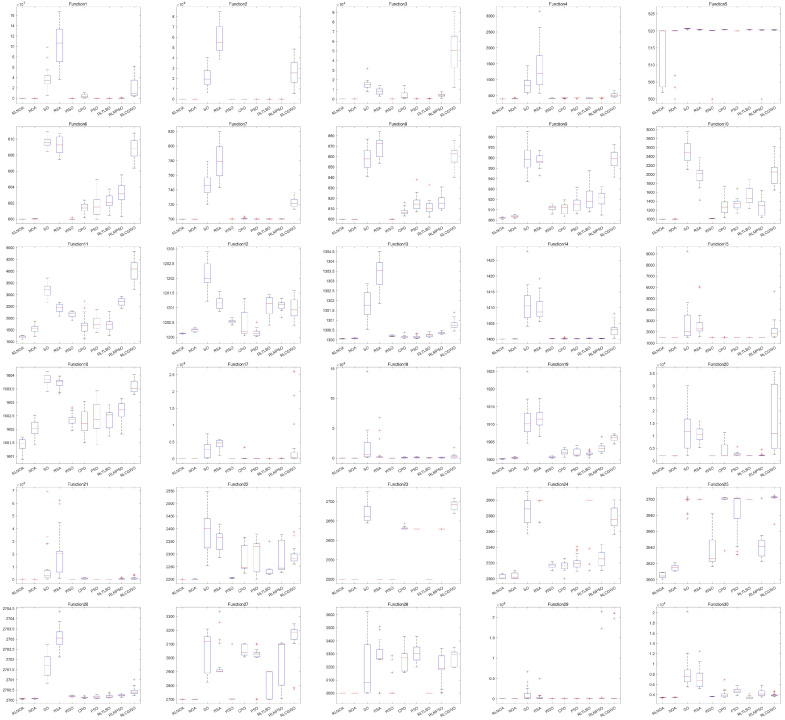
The box plots of different algorithms applied to the CEC-2014 test suite.

Figure 4 illustrates the convergence curves of different algorithms applied to the CEC-2014 test suite. It can be seen from the figure that the convergence speed of the RLNOA is generally not the fastest. This is primarily because RL allows the population to learn the best exploitation strategy in the current state during the early stages of the search. As a result, the convergence speed of the RLNOA in the early stages is not the best, but its overall convergence performance remains competitive compared to the other algorithms. Moreover, due to RL combined with a dynamic exploration mechanism, the RLNOA can avoid local optima. Particularly for functions F5, F6, F8, F9, F10, F11, and F16, the RLNOA achieves better convergence results. Figure 5 shows the box plots of different algorithms applied to the CEC-2014 test suite, where the RLNOA achieves superior results.

### 4.3. Comparison over CEC-2017

In this experiment, the ability of the RLNOA to solve the optimization problems within the CEC-2017 test suite is evaluated, with the results presented in Table 3. The CEC-2017 benchmark suite consists of 30 test functions, categorized into unimodal functions, basic multimodal functions, expanded multimodal functions, hybrid functions, and composition functions. These categories encompass a wide range of optimization challenges, assessing the ability of algorithms to locate global optima, avoid local optima, and effectively explore the search space. It is also noted that the CEC-2017-F2 function was excluded from the test suite due to its unstable behavior.

**Figure 6:**
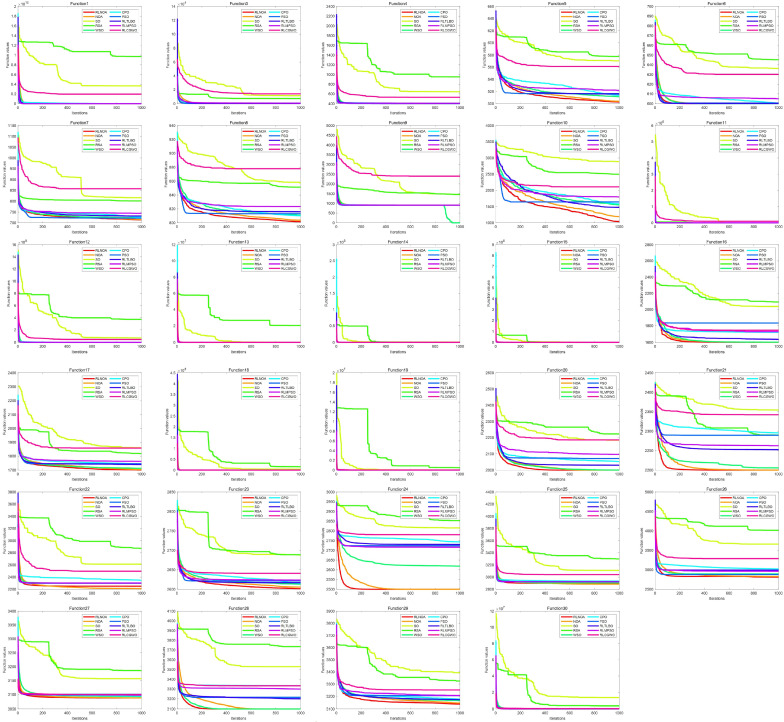
The convergence curves of different algorithms applied to the CEC-2017 test suite.

**Figure 7:**
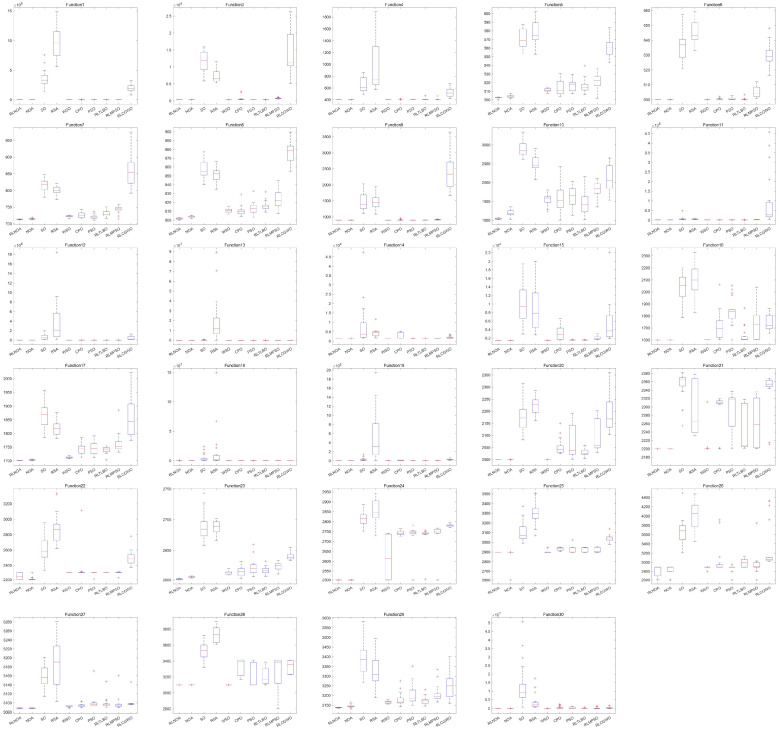
The box plots of different algorithms applied to the CEC-2017 test suite.

As shown in Table 3, the proposed RLNOA obtained the most optimal values, ranking first overall. For the five functions where the RLNOA did not achieve the optimal value, it ranked second. The RSA performed the worst when applied to the CEC-2017 test suite, ranking last. Figure 6 illustrates the convergence curves of the different algorithms applied to the CEC-2017 test suite. It can be seen that the RLNOA converges faster than other methods for functions such as F16, F20, and F24. In most cases, its convergence speed surpasses that of algorithms such as the SO and RSA. Figure 7 presents the box plots of different algorithms on the CEC-2017 test suite, indicating that the RLNOA consistently achieved superior results.

### 4.4. Comparison over CEC-2020

To verify the effectiveness of the proposed algorithm for problems with enhanced complexity and realism, we performed optimization experiments on the CEC-2020 test suite. This suite comprises 10 benchmark functions and places a greater emphasis on dynamic and noisy functions, reflecting the evolving nature of real-world problems. Such a focus allows for a comprehensive evaluation of an algorithm’s performance under more variable and unpredictable conditions.

**Figure 8:**
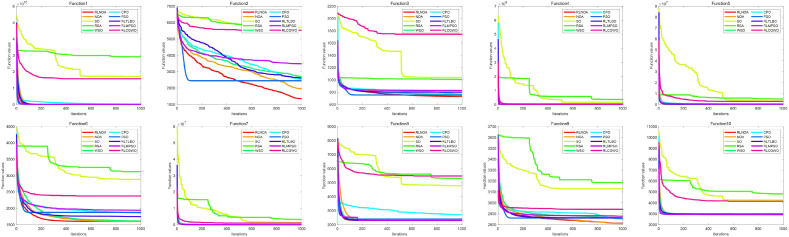
The convergence curves of different algorithms applied to the CEC-2020 test suite.

**Figure 9:**
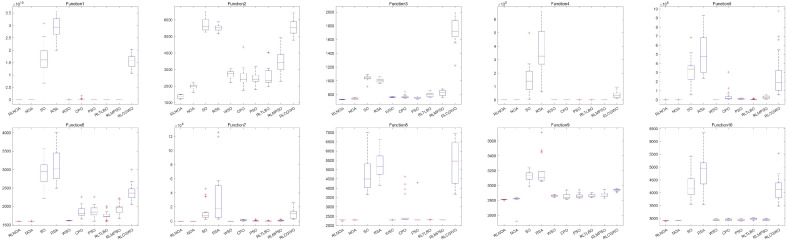
The box plots of different algorithms applied to the CEC-2020 test suite.

Table 4 presents the results of different algorithms applied to the CEC-2020 test suite. It can be seen that the RLNOA ranks first in eight out of the 10 functions. For functions F4 and F9, the RLNOA ranks second, but the gap between its results and the first-ranked results is minimal. The average ranking and final ranking of each algorithm across all test functions are shown in the last two rows of Table 4. The RLNOA performs the best, with an average ranking of 1.2222 and a final ranking of 1. Figure 8 and Figure 9, respectively, show the convergence curves and box plots of different algorithms applied to the CEC-2020 test suite. These figures confirm that the RLNOA consistently demonstrates superior performance across all benchmark functions.

### 4.5. Analysis of the Q-Table

**Figure 10:**
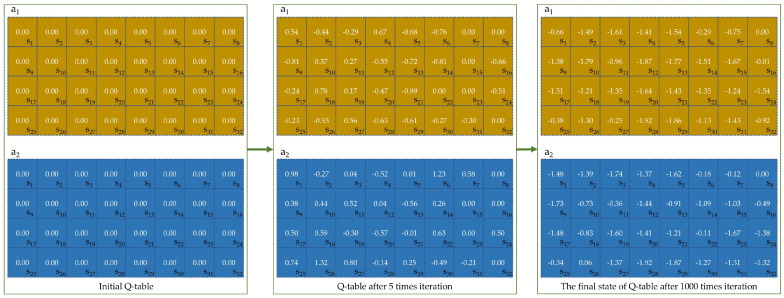
Q-table update process for the F1 function.

We take test function F1 in CEC2014 as an example to illustrate the Q-table update process. As shown in Figure 10, the state $\left[s_{1},s_{2},\ldots ,s_{32}\right]$ represents different stages of the relative changes in local diversity and fitness values. Actions $a_{1}$ and $a_{2}$ represent the storage strategy and recovery strategy, respectively. The Q-table is initialized as a zero matrix. After five iterations, the Q-values for different states in the Q-table change. The individual will execute the action corresponding to the highest Q-value in the current state. For example, the Q-value of the storage strategy (action $a_{1}$) in state $s_{26}$ is −0.93, which is much lower than the Q-value of the recovery strategy (action $a_{2}$), which is 1.32. When the individual is in state $s_{26}$ during the next iteration, it will execute the recovery strategy. In the later stages of the optimization process, when the population has converged close to the global optimum, selecting any exploitation strategy is unlikely to yield better results. As a result, almost all Q-values in the Q-table become negative.

### 4.6. Analysis of the RLNOA’s Parameters

The RLNOA contains some parameters that affect its performance, similar to other metaheuristic algorithms. Here are some suggestions for selecting and tuning these parameters.

(1) Population size *NP*

A larger population size generally allows for better exploration of the solution space, as more diverse solutions are maintained. However, increasing the population size typically leads to higher computational costs, as more solutions need to be evaluated during each iteration. This can slow down the algorithm, especially for complex or large-scale problems. Therefore, population sizes often range from a few dozen to several hundred individuals, depending on the problem’s complexity and the specific metaheuristic used. Reasonable values are within [20, 200].

(2) Maximum iterations $T_{\max}$

More iterations allow the algorithm to refine and improve solutions gradually. However, there may be diminishing returns after a certain number of iterations, where further improvements become minimal. Reasonable values are within [100, 2000].

(3) Learning rate α

This parameter determines how much new information overrides old information. A high learning rate means the agent learns quickly, but it may also make the learning process unstable. A low learning rate ensures stability but can slow down the learning process. Reasonable values are within [0.1, 0.9].

(4) Discount factor γ

This factor determines the importance of future rewards. A value close to 0 makes the agent prioritize immediate rewards, while a value close to 1 encourages the agent to consider long-term rewards. The discount factor helps balance immediate versus future gains, influencing the agent’s overall strategy. Reasonable values are within [0.1, 0.9].

We also studied the sensitivity of the RLNOA’s partial parameters. This analysis helps determine the impact of small changes in these parameters on the performance of the proposed algorithm. The functions used in these experiments are F1, F4, F17, and F23 from CEC-2014, which represent unimodal, multimodal, hybrid, and composite functions, respectively. The maximum number of iterations is set to 1000. The population size is set to 100. The average (Ave) of fitness values from 30 independent runs is used to acquire the sensitivity analysis results. Experimental results are as follows:

- Global exploration threshold $P_{rp}$: to verify the effect of $P_{rp}$ on the efficiency of the RLNOA, experiments are performed for several values of $P_{rp}$, taken as 0.2, 0.4, 0.6, and 0.8, while other parameters are unchanged. As shown in Table 5, the RLNOA is insensitive to this parameter. The results of F17 indicate that the RLNOA performs best when $P_{rp}=0.2$ is set to a specific value.
- Control parameter δ: experiments are performed for several values of δ, taken as 0.05, 0.1, 0.2, and 0.5, while other parameters are unchanged. As shown in Table 6, the RLNOA is not sensitive to small changes in the parameter δ.
- Number of near neighbors k: Table 7 shows the results of the RLNOA with different values for the parameter k. It is evident from Table 7 that the RLNOA is not sensitive to small changes in the parameter k.
- Control parameter ζ: to explore the sensitivity of the RLNOA to the parameter ζ, experiments are caried out for different values of ζ, as shown in Table 8. It is apparent that the RLNOA is sensitive to ζ. This is primarily because ζ controls the variation trend of the exploration sub-population during the optimization process. Table 8 also shows that the RLNOA acquires the best results when the value of ζ is set to 1.

## 5. Conclusions

In this paper, we propose an RL-based bi-population nutcracker optimizer algorithm. We developed a bi-population mechanism that uses fitness ranking to separate the raw population into exploration and exploitation sub-populations at the start of each iteration. The exploration sub-population, comprising individuals with lower fitness, employs a foraging strategy based on ROBL to maintain diversity. The exploitation sub-population includes two strategies, storage and recovery, with the selection of strategy controlled by Q-learning in RL. Experiments were conducted on the CEC-2014, CEC-2017, and CEC-2020 benchmark suites. The results, including optimization performance, convergence curves, and box plots, demonstrate that the proposed algorithm outperforms nine other comparative algorithms.

However, the proposed algorithm still has limitations. First, the exploration strategy needs to be further enhanced to improve the algorithm’s ability to escape local optima. Second, there is some redundancy in boundary states within the Q-learning process. In the future, based on the existing RLNOA framework, the development of search strategies and the encoding of states will be further studied to improve optimization performance. Additionally, complex engineering applications will be introduced to test future work.

## Figures and Tables

**Table 1:** The common parameters of the experimental algorithms.

Algorithm	Specifications	Population Size *NP*
RLNOA	Learning rate α=0.5, discount factor γ=0.5, $P_{rp}=0.2,\;$ δ=0.05, k=20, ζ=1	100
NOA	$P_{a1}$ $\;\mathrm{decreases}\;\mathrm{linearly}\;\mathrm{from}\;2\;\mathrm{to}\;0,\;Pa_{2}=0.2,\;$ $P_{rp}=0.2,\;$ δ=0.05	100
SO	$c_{1}=0.5,\;$ $c_{2}=0.05,\;$ $c_{3}=2$	100
RSA	α=0.1, β=0.005	100
CPO	The number of cycles T=2, the convergence rate α=0.2, $T_{f}=0.8,\;$ $N_{\min}=20$	100
GWO	Convergence constant a decreases linearly from 2 to 0	100
PSO	ω=1, $c_{1}=1.5,\;$ $c_{2}=2$	100
RLTLBO	Learning rate α=0.5, discount factor γ=0.5	33 because this algorithm has three main stages
RLMPSO	Learning rate α=0.5, discount factor γ=0.5, ω=0.9 for exploration, ω=0.4$\;\mathrm{for}\;\mathrm{convergence},\;c_{1}=2.5$$\;\mathrm{for}\;\mathrm{exploration},\;c_{2}=0.5$$\;\mathrm{for}\;\mathrm{convergence},\;c_{1}=0.5$$\;\mathrm{for}\;\mathrm{exploration},\;c_{2}=2.5$$\;\mathrm{for}\;\mathrm{convergence},\;\left[V_{\min},V_{\max}\right]$ is set as 0.2 of search range	100
RLCGWO	Learning rate α=0.5, discount factor γ=0.5, scaling factor e=0.5, the minimum learning probability a=0.1, the maximum learning probability b=0.5	100

**Table 2:** Results of the CEC-2014 test suite.

Fun	Metrics	RLNOA	NOA	SO	RSA	CPO	GWO	PSO	RLTLBO	RLMPSO	RLCGWO
F1	Ave	1.00 × 10^2^	1.04 × 10^2^	3.86 × 10^7^	1.02 × 10^8^	1.64 × 10^2^	4.57 × 10^6^	1.24 × 10^4^	2.39 × 10^4^	3.28 × 10^5^	1.86 × 10^7^
	Std	4.65 × 10^−3^	2.55 × 10^0^	2.05 × 10^7^	4.09 × 10^7^	4.73 × 10^1^	3.34 × 10^6^	1.33 × 10^4^	3.63 × 10^4^	2.44 × 10^5^	1.79 × 10^7^
	Rank	1	2	9	10	3	7	4	5	6	8
F2	Ave	2.00 × 10^2^	2.00 × 10^2^	2.16 × 10^9^	5.89 × 10^9^	2.00 × 10^2^	6.15 × 10^3^	1.10 × 10^3^	5.83 × 10^2^	2.04 × 10^3^	2.60 × 10^9^
	Std	2.61 × 10^−6^	1.15 × 10^−3^	9.40 × 10^8^	1.50 × 10^9^	1.03 × 10^−5^	4.49 × 10^3^	1.17 × 10^3^	6.06 × 10^2^	2.90 × 10^3^	1.26 × 10^9^
	Rank	1	3	8	10	2	7	5	4	6	9
F3	Ave	3.00 × 10^2^	3.00 × 10^2^	1.54 × 10^4^	8.05 × 10^3^	3.00 × 10^2^	4.01 × 10^3^	3.39 × 10^2^	3.89 × 10^2^	4.27 × 10^3^	4.97 × 10^4^
	Std	1.52 × 10^−8^	1.60 × 10^−6^	4.95 × 10^3^	3.32 × 10^3^	1.12 × 10^−8^	3.55 × 10^3^	6.59 × 10^1^	1.18 × 10^2^	1.70 × 10^3^	2.18 × 10^4^
	Rank	2	3	9	8	1	6	4	5	7	10
F4	Ave	4.00 × 10^2^	4.05 × 10^2^	8.49 × 10^2^	1.37 × 10^3^	4.09 × 10^2^	4.29 × 10^2^	4.28 × 10^2^	4.16 × 10^2^	4.31 × 10^2^	5.15 × 10^2^
	Std	3.68 × 10^−3^	1.14 × 10^1^	2.81 × 10^2^	6.97 × 10^2^	1.51 × 10^1^	1.17 × 10^1^	1.34 × 10^1^	1.63 × 10^1^	8.74 × 10^0^	5.83 × 10^1^
	Rank	1	2	9	10	3	6	5	4	7	8
F5	Ave	5.13 × 10^2^	5.18 × 10^2^	5.21 × 10^2^	5.20 × 10^2^	5.17 × 10^2^	5.20 × 10^2^	5.20 × 10^2^	5.20 × 10^2^	5.19 × 10^2^	5.20 × 10^2^
	Std	8.38 × 10^0^	6.18 × 10^0^	1.16 × 10^−1^	8.23 × 10^−2^	7.38 × 10^0^	5.93 × 10^−2^	1.06 × 10^−3^	6.58 × 10^−2^	4.55 × 10^0^	1.13 × 10^−1^
	Rank	1	3	10	9	2	7	5	8	4	6
F6	Ave	6.00 × 10^2^	6.00 × 10^2^	6.10 × 10^2^	6.09 × 10^2^	6.00 × 10^2^	6.01 × 10^2^	6.02 × 10^2^	6.02 × 10^2^	6.03 × 10^2^	6.09 × 10^2^
	Std	6.70 × 10^−6^	1.89 × 10^−2^	6.27 × 10^−1^	1.09 × 10^0^	4.34 × 10^−2^	6.05 × 10^−1^	1.47 × 10^0^	9.28 × 10^−1^	1.34 × 10^0^	1.19 × 10^0^
	Rank	1	3	10	9	2	4	5	6	7	8
F7	Ave	7.00 × 10^2^	7.00 × 10^2^	7.48 × 10^2^	7.79 × 10^2^	7.00 × 10^2^	7.01 × 10^2^	7.00 × 10^2^	7.00 × 10^2^	7.00 × 10^2^	7.23 × 10^2^
	Std	8.66 × 10^−3^	2.32 × 10^−2^	1.69 × 10^1^	2.48 × 10^1^	3.57 × 10^−2^	1.03 × 10^0^	7.46 × 10^−2^	8.26 × 10^−2^	8.76 × 10^−2^	7.51 × 10^0^
	Rank	1	2	9	10	3	7	4	5	6	8
F8	Ave	8.00 × 10^2^	8.00 × 10^2^	8.58 × 10^2^	8.69 × 10^2^	8.00 × 10^2^	8.07 × 10^2^	8.15 × 10^2^	8.11 × 10^2^	8.16 × 10^2^	8.60 × 10^2^
	Std	0.00 × 10^0^	3.58 × 10^−13^	1.10 × 10^1^	9.38 × 10^0^	2.83 × 10^−10^	3.19 × 10^0^	7.35 × 10^0^	6.43 × 10^0^	6.83 × 10^0^	9.87 × 10^0^
	Rank	1	2	8	10	3	4	6	5	7	9
F9	Ave	9.02 × 10^2^	9.04 × 10^2^	9.61 × 10^2^	9.58 × 10^2^	9.12 × 10^2^	9.11 × 10^2^	9.15 × 10^2^	9.22 × 10^2^	9.21 × 10^2^	9.59 × 10^2^
	Std	6.20 × 10^−1^	1.14 × 10^0^	1.27 × 10^1^	5.78 × 10^0^	2.46 × 10^0^	4.78 × 10^0^	7.19 × 10^0^	1.26 × 10^1^	7.73 × 10^0^	8.83 × 10^0^
	Rank	1	2	10	8	4	3	5	7	6	9
F10	Ave	1.00 × 10^3^	1.00 × 10^3^	2.51 × 10^3^	1.97 × 10^3^	1.01 × 10^3^	1.30 × 10^3^	1.35 × 10^3^	1.52 × 10^3^	1.29 × 10^3^	2.01 × 10^3^
	Std	5.16 × 10^−2^	9.51 × 10^−1^	2.37 × 10^2^	2.07 × 10^2^	3.48 × 10^0^	2.15 × 10^2^	1.25 × 10^2^	1.93 × 10^2^	1.88 × 10^2^	2.48 × 10^2^
	Rank	1	2	10	8	3	5	6	7	4	9
F11	Ave	1.22 × 10^3^	1.56 × 10^3^	3.18 × 10^3^	2.43 × 10^3^	2.16 × 10^3^	1.72 × 10^3^	1.80 × 10^3^	1.73 × 10^3^	2.70 × 10^3^	4.04 × 10^3^
	Std	4.64 × 10^1^	1.65 × 10^2^	2.95 × 10^2^	1.80 × 10^2^	1.26 × 10^2^	3.94 × 10^2^	2.84 × 10^2^	2.64 × 10^2^	1.41 × 10^2^	4.54 × 10^2^
	Rank	1	2	9	7	6	3	5	4	8	10
F12	Ave	1.20 × 10^3^	1.20 × 10^3^	1.20 × 10^3^	1.20 × 10^3^	1.20 × 10^3^	1.20 × 10^3^	1.20 × 10^3^	1.20 × 10^3^	1.20 × 10^3^	1.20 × 10^3^
	Std	1.74 × 10^−2^	5.36 × 10^−2^	4.70 × 10^−1^	2.00 × 10^−1^	6.69 × 10^−2^	4.50 × 10^−1^	1.23 × 10^−1^	3.44 × 10^−1^	1.55 × 10^−1^	3.60 × 10^−1^
	Rank	1	3	10	9	5	4	2	7	8	6
F13	Ave	1.30 × 10^3^	1.30 × 10^3^	1.30 × 10^3^	1.30 × 10^3^	1.30 × 10^3^	1.30 × 10^3^	1.30 × 10^3^	1.30 × 10^3^	1.30 × 10^3^	1.30 × 10^3^
	Std	8.47 × 10^−3^	1.91 × 10^−2^	6.58 × 10^−1^	8.19 × 10^−1^	2.70 × 10^−2^	6.50 × 10^−2^	7.76 × 10^−2^	8.02 × 10^−2^	5.51 × 10^−2^	2.29 × 10^−1^
	Rank	1	2	9	10	5	4	3	6	7	8
F14	Ave	1.40 × 10^3^	1.40 × 10^3^	1.41 × 10^3^	1.41 × 10^3^	1.40 × 10^3^	1.40 × 10^3^	1.40 × 10^3^	1.40 × 10^3^	1.40 × 10^3^	1.40 × 10^3^
	Std	1.29 × 10^−2^	3.13 × 10^−2^	5.57 × 10^0^	3.50 × 10^0^	3.96 × 10^−2^	1.66 × 10^−1^	5.65 × 10^−2^	1.02 × 10^−1^	4.79 × 10^−2^	1.93 × 10^0^
	Rank	1	2	10	9	4	5	3	7	6	8
F15	Ave	1.50 × 10^3^	1.50 × 10^3^	2.77 × 10^3^	2.68 × 10^3^	1.50 × 10^3^	1.50 × 10^3^	1.50 × 10^3^	1.50 × 10^3^	1.50 × 10^3^	2.14 × 10^3^
	Std	8.24 × 10^−2^	1.35 × 10^−1^	1.83 × 10^3^	1.25 × 10^3^	2.30 × 10^−1^	6.79 × 10^−1^	3.66 × 10^−1^	4.93 × 10^−1^	9.82 × 10^−1^	9.17 × 10^2^
	Rank	1	2	10	9	5	4	3	6	7	8
F16	Ave	1.60 × 10^3^	1.60 × 10^3^	1.60 × 10^3^	1.60 × 10^3^	1.60 × 10^3^	1.60 × 10^3^	1.60 × 10^3^	1.60 × 10^3^	1.60 × 10^3^	1.60 × 10^3^
	Std	2.10 × 10^−1^	2.95 × 10^−1^	1.86 × 10^−1^	1.87 × 10^−1^	2.46 × 10^−1^	4.42 × 10^−1^	5.19 × 10^−1^	3.45 × 10^−1^	4.04 × 10^−1^	2.30 × 10^−1^
	Rank	1	2	10	9	4	3	6	5	7	8
F17	Ave	1.71 × 10^3^	1.73 × 10^3^	2.74 × 10^5^	4.34 × 10^5^	1.79 × 10^3^	4.06 × 10^4^	4.15 × 10^3^	2.57 × 10^3^	7.96 × 10^3^	4.44 × 10^5^
	Std	2.72 × 10^0^	1.17 × 10^1^	2.38 × 10^5^	1.39 × 10^5^	2.59 × 10^1^	1.04 × 10^5^	2.11 × 10^3^	6.71 × 10^2^	3.88 × 10^3^	8.66 × 10^5^
	Rank	1	2	8	9	3	7	5	4	6	10
F18	Ave	1.80 × 10^3^	1.80 × 10^3^	1.95 × 10^5^	8.88 × 10^4^	1.80 × 10^3^	9.13 × 10^3^	1.25 × 10^4^	4.15 × 10^3^	9.55 × 10^3^	3.19 × 10^4^
	Std	1.66 × 10^−1^	5.94 × 10^−1^	3.26 × 10^5^	1.83 × 10^5^	7.55 × 10^−1^	6.34 × 10^3^	7.24 × 10^3^	1.78 × 10^3^	6.03 × 10^3^	3.89 × 10^4^
	Rank	1	2	10	9	3	5	7	4	6	8
F19	Ave	1.90 × 10^3^	1.90 × 10^3^	1.91 × 10^3^	1.91 × 10^3^	1.90 × 10^3^	1.90 × 10^3^	1.90 × 10^3^	1.90 × 10^3^	1.90 × 10^3^	1.91 × 10^3^
	Std	5.49 × 10^−2^	1.78 × 10^−1^	4.63 × 10^0^	2.64 × 10^0^	1.98 × 10^−1^	7.61 × 10^−1^	1.02 × 10^0^	5.08 × 10^−1^	1.15 × 10^0^	7.90 × 10^−1^
	Rank	1	2	9	10	3	5	6	4	7	8
F20	Ave	2.00 × 10^3^	2.00 × 10^3^	1.22 × 10^4^	1.05 × 10^4^	2.00 × 10^3^	4.80 × 10^3^	2.67 × 10^3^	2.11 × 10^3^	2.34 × 10^3^	1.62 × 10^4^
	Std	4.59 × 10^−2^	1.65 × 10^−1^	7.65 × 10^3^	3.22 × 10^3^	3.20 × 10^−1^	3.80 × 10^3^	8.27 × 10^2^	3.41 × 10^1^	5.31 × 10^2^	1.26 × 10^4^
	Rank	1	2	9	8	3	7	6	4	5	10
F21	Ave	2.10 × 10^3^	2.10 × 10^3^	1.02 × 10^5^	2.04 × 10^5^	2.11 × 10^3^	8.61 × 10^3^	2.27 × 10^3^	2.26 × 10^3^	6.38 × 10^3^	1.21 × 10^4^
	Std	1.49 × 10^−1^	5.73 × 10^−1^	1.72 × 10^5^	1.76 × 10^5^	1.96 × 10^0^	4.53 × 10^3^	1.29 × 10^2^	9.87 × 10^1^	4.71 × 10^3^	1.13 × 10^4^
	Rank	1	2	9	10	3	7	5	4	6	8
F22	Ave	2.20 × 10^3^	2.20 × 10^3^	2.39 × 10^3^	2.35 × 10^3^	2.21 × 10^3^	2.28 × 10^3^	2.29 × 10^3^	2.23 × 10^3^	2.28 × 10^3^	2.29 × 10^3^
	Std	6.58 × 10^−2^	6.00 × 10^−1^	8.30 × 10^1^	4.06 × 10^1^	1.22 × 10^0^	5.28 × 10^1^	6.40 × 10^1^	2.86 × 10^1^	5.90 × 10^1^	3.40 × 10^1^
	Rank	1	2	10	9	3	6	7	4	5	8
F23	Ave	2.50 × 10^3^	2.50 × 10^3^	2.67 × 10^3^	2.50 × 10^3^	2.50 × 10^3^	2.63 × 10^3^	2.63 × 10^3^	2.50 × 10^3^	2.63 × 10^3^	2.69 × 10^3^
	Std	0.00 × 10^0^	0.00 × 10^0^	2.48 × 10^1^	0.00 × 10^0^	0.00 × 10^0^	3.56 × 10^0^	2.95 × 10^−13^	0.00 × 10^0^	2.77 × 10^−7^	1.17 × 10^1^
	Rank	1	2	9	3	4	8	6	5	7	10
F24	Ave	2.50 × 10^3^	2.50 × 10^3^	2.59 × 10^3^	2.60 × 10^3^	2.52 × 10^3^	2.52 × 10^3^	2.52 × 10^3^	2.59 × 10^3^	2.53 × 10^3^	2.58 × 10^3^
	Std	2.76 × 10^0^	3.85 × 10^0^	1.62 × 10^1^	8.64 × 10^0^	2.98 × 10^0^	5.84 × 10^0^	8.79 × 10^0^	2.88 × 10^1^	9.68 × 10^0^	1.37 × 10^1^
	Rank	1	2	8	10	4	3	5	9	6	7
F25	Ave	2.61 × 10^3^	2.62 × 10^3^	2.70 × 10^3^	2.70 × 10^3^	2.64 × 10^3^	2.70 × 10^3^	2.68 × 10^3^	2.70 × 10^3^	2.64 × 10^3^	2.70 × 10^3^
	Std	3.29 × 10^0^	3.40 × 10^0^	7.49 × 10^0^	1.10 × 10^−2^	2.25 × 10^1^	1.46 × 10^1^	2.73 × 10^1^	0.00 × 10^0^	1.74 × 10^1^	7.56 × 10^0^
	Rank	1	2	6	8	3	7	5	9	4	10
F26	Ave	2.70 × 10^3^	2.70 × 10^3^	2.70 × 10^3^	2.70 × 10^3^	2.70 × 10^3^	2.70 × 10^3^	2.70 × 10^3^	2.70 × 10^3^	2.70 × 10^3^	2.70 × 10^3^
	Std	9.37 × 10^−3^	1.53 × 10^−2^	5.68 × 10^−1^	5.20 × 10^−1^	2.72 × 10^−2^	3.39 × 10^−2^	6.53 × 10^−2^	7.06 × 10^−2^	4.19 × 10^−2^	1.82 × 10^−1^
	Rank	1	2	9	10	6	3	4	5	7	8
F27	Ave	2.70 × 10^3^	2.70 × 10^3^	3.04 × 10^3^	2.96 × 10^3^	2.72 × 10^3^	3.05 × 10^3^	2.99 × 10^3^	2.81 × 10^3^	2.97 × 10^3^	3.14 × 10^3^
	Std	2.19 × 10^−1^	3.35 × 10^−1^	1.44 × 10^2^	1.33 × 10^2^	8.89 × 10^1^	3.79 × 10^1^	1.27 × 10^2^	1.01 × 10^2^	1.73 × 10^2^	1.29 × 10^2^
	Rank	1	2	8	5	3	9	7	4	6	10
F28	Ave	3.00 × 10^3^	3.00 × 10^3^	3.19 × 10^3^	3.27 × 10^3^	3.04 × 10^3^	3.26 × 10^3^	3.31 × 10^3^	3.00 × 10^3^	3.19 × 10^3^	3.26 × 10^3^
	Std	0.00 × 10^0^	0.00 × 10^0^	2.19 × 10^2^	1.40 × 10^2^	8.85 × 10^1^	8.53 × 10^1^	7.12 × 10^1^	0.00 × 10^0^	1.08 × 10^2^	5.94 × 10^1^
	Rank	1	2	5	9	4	7	10	3	6	8
F29	Ave	3.05 × 10^3^	3.10 × 10^3^	1.14 × 10^5^	4.50 × 10^4^	3.12 × 10^3^	3.68 × 10^3^	3.24 × 10^3^	3.15 × 10^3^	1.98 × 10^5^	4.18 × 10^5^
	Std	6.18 × 10^0^	1.85 × 10^1^	1.75 × 10^5^	1.10 × 10^5^	1.22 × 10^1^	5.20 × 10^2^	9.86 × 10^1^	1.56 × 10^2^	5.98 × 10^5^	8.48 × 10^5^
	Rank	1	2	8	7	3	6	5	4	9	10
F30	Ave	3.45 × 10^3^	3.49 × 10^3^	8.35 × 10^3^	7.21 × 10^3^	3.64 × 10^3^	4.10 × 10^3^	4.74 × 10^3^	3.37 × 10^3^	4.34 × 10^3^	3.92 × 10^3^
	Std	4.79 × 10^1^	1.63 × 10^1^	3.30 × 10^3^	2.05 × 10^3^	3.27 × 10^1^	7.90 × 10^2^	4.97 × 10^2^	2.72 × 10^2^	7.15 × 10^2^	2.90 × 10^2^
	Rank	2	3	10	9	4	6	8	1	7	5
Mean	Ranking	1.0345	2.1724	8.8966	8.6897	3.4483	5.4828	5.1379	5.3103	6.3103	8.5172
Final	Rank	1	2	10	9	3	6	4	5	7	8

**Table 3:** Results of the CEC-2017 test suite.

Fun	Metrics	RLNOA	NOA	SO	RSA	CPO	GWO	PSO	RLTLBO	RLMPSO	RLCGWO
F1	Ave	1.00 × 10^2^	1.00 × 10^2^	3.65 × 10^9^	9.70 × 10^9^	1.00 × 10^2^	1.30 × 10^6^	1.25 × 10^3^	2.12 × 10^3^	3.10 × 10^3^	1.95 × 10^9^
	Std	8.32 × 10^−5^	1.59 × 10^−2^	1.44 × 10^9^	2.68 × 10^9^	3.70 × 10^−4^	4.96 × 10^6^	2.07 × 10^3^	2.45 × 10^3^	3.34 × 10^3^	6.61 × 10^8^
	Rank	1	3	9	10	2	7	4	5	6	8
F3	Ave	3.00 × 10^2^	3.00 × 10^2^	1.14 × 10^4^	7.33 × 10^3^	3.00 × 10^2^	7.61 × 10^2^	3.00 × 10^2^	3.00 × 10^2^	7.22 × 10^2^	1.42 × 10^4^
	Std	1.32 × 10^−11^	5.52 × 10^−8^	3.10 × 10^3^	2.01 × 10^3^	9.79 × 10^−7^	8.18 × 10^2^	4.12 × 10^−14^	3.04 × 10^−9^	1.65 × 10^2^	6.44 × 10^3^
	Rank	2	4	9	8	5	7	1	3	6	10
F4	Ave	4.00 × 10^2^	4.00 × 10^2^	6.44 × 10^2^	9.52 × 10^2^	4.01 × 10^2^	4.08 × 10^2^	4.01 × 10^2^	4.07 × 10^2^	4.08 × 10^2^	5.30 × 10^2^
	Std	8.57 × 10^−6^	7.44 × 10^−3^	1.22 × 10^2^	4.15 × 10^2^	4.06 × 10^−1^	2.34 × 10^0^	7.61 × 10^−1^	1.46 × 10^1^	1.36 × 10^1^	8.14 × 10^1^
	Rank	1	2	9	10	3	6	4	5	7	8
F5	Ave	5.03 × 10^2^	5.04 × 10^2^	5.70 × 10^2^	5.78 × 10^2^	5.12 × 10^2^	5.14 × 10^2^	5.17 × 10^2^	5.16 × 10^2^	5.22 × 10^2^	5.61 × 10^2^
	Std	7.16 × 10^−1^	1.42 × 10^0^	1.13 × 10^1^	1.38 × 10^1^	1.70 × 10^0^	8.36 × 10^0^	6.65 × 10^0^	7.73 × 10^0^	8.66 × 10^0^	1.07 × 10^1^
	Rank	1	2	9	10	3	4	6	5	7	8
F6	Ave	6.00 × 10^2^	6.00 × 10^2^	6.36 × 10^2^	6.45 × 10^2^	6.00 × 10^2^	6.00 × 10^2^	6.01 × 10^2^	6.00 × 10^2^	6.05 × 10^2^	6.30 × 10^2^
	Std	4.16 × 10^−12^	4.59 × 10^−8^	9.52 × 10^0^	7.07 × 10^0^	1.04 × 10^−6^	5.11 × 10^−1^	7.29 × 10^−1^	7.53 × 10^−1^	3.59 × 10^0^	7.44 × 10^0^
	Rank	1	2	9	10	3	5	6	4	7	8
F7	Ave	7.14 × 10^2^	7.15 × 10^2^	8.16 × 10^2^	8.01 × 10^2^	7.23 × 10^2^	7.26 × 10^2^	7.21 × 10^2^	7.31 × 10^2^	7.44 × 10^2^	8.58 × 10^2^
	Std	6.13 × 10^−1^	1.58 × 10^0^	1.82 × 10^1^	1.26 × 10^1^	2.83 × 10^0^	9.37 × 10^0^	6.15 × 10^0^	9.14 × 10^0^	1.08 × 10^1^	4.68 × 10^1^
	Rank	1	2	9	8	4	5	3	6	7	10
F8	Ave	8.02 × 10^2^	8.04 × 10^2^	8.58 × 10^2^	8.51 × 10^2^	8.11 × 10^2^	8.10 × 10^2^	8.13 × 10^2^	8.16 × 10^2^	8.23 × 10^2^	8.78 × 10^2^
	Std	7.86 × 10^−1^	9.54 × 10^−1^	9.68 × 10^0^	7.96 × 10^0^	2.33 × 10^0^	5.53 × 10^0^	6.70 × 10^0^	5.15 × 10^0^	9.85 × 10^0^	1.21 × 10^1^
	Rank	1	2	9	8	4	3	5	6	7	10
F9	Ave	9.00 × 10^2^	9.00 × 10^2^	1.46 × 10^3^	1.46 × 10^3^	9.00 × 10^2^	9.06 × 10^2^	9.00 × 10^2^	9.02 × 10^2^	9.08 × 10^2^	2.40 × 10^3^
	Std	0.00 × 10^0^	2.61 × 10^−14^	2.65 × 10^2^	2.16 × 10^2^	0.00 × 10^0^	1.43 × 10^1^	4.52 × 10^−14^	1.48 × 10^0^	7.89 × 10^0^	5.80 × 10^2^
	Rank	1	2	8	9	3	6	4	5	7	10
F10	Ave	1.04 × 10^3^	1.19 × 10^3^	2.89 × 10^3^	2.50 × 10^3^	1.55 × 10^3^	1.58 × 10^3^	1.64 × 10^3^	1.47 × 10^3^	1.80 × 10^3^	2.11 × 10^3^
	Std	1.95 × 10^1^	8.33 × 10^1^	2.00 × 10^2^	1.85 × 10^2^	1.44 × 10^2^	3.29 × 10^2^	2.47 × 10^2^	3.29 × 10^2^	2.30 × 10^2^	3.47 × 10^2^
	Rank	1	2	10	9	4	5	6	3	7	8
F11	Ave	1.10 × 10^3^	1.10 × 10^3^	6.78 × 10^3^	4.83 × 10^3^	1.10 × 10^3^	1.12 × 10^3^	1.11 × 10^3^	1.11 × 10^3^	1.72 × 10^3^	9.36 × 10^4^
	Std	2.58 × 10^−1^	1.11 × 10^0^	1.02 × 10^4^	2.47 × 10^3^	4.60 × 10^−1^	1.74 × 10^1^	6.89 × 10^0^	9.19 × 10^0^	4.22 × 10^2^	1.38 × 10^5^
	Rank	1	2	9	8	3	6	4	5	7	10
F12	Ave	1.24 × 10^3^	1.36 × 10^3^	7.05 × 10^7^	3.70 × 10^8^	1.55 × 10^3^	6.79 × 10^5^	1.19 × 10^4^	1.16 × 10^4^	2.81 × 10^5^	4.66 × 10^7^
	Std	1.38 × 10^1^	4.92 × 10^1^	6.17 × 10^7^	4.42 × 10^8^	8.08 × 10^1^	9.50 × 10^5^	9.58 × 10^3^	7.96 × 10^3^	1.10 × 10^6^	4.11 × 10^7^
	Rank	1	2	9	10	3	7	5	4	6	8
F13	Ave	1.30 × 10^3^	1.31 × 10^3^	2.15 × 10^5^	2.03 × 10^7^	1.31 × 10^3^	9.55 × 10^3^	7.69 × 10^3^	3.28 × 10^3^	1.25 × 10^4^	3.91 × 10^4^
	Std	7.95 × 10^−1^	1.35 × 10^0^	2.41 × 10^5^	2.30 × 10^7^	2.99 × 10^0^	4.31 × 10^3^	5.51 × 10^3^	1.77 × 10^3^	8.14 × 10^3^	2.96 × 10^4^
	Rank	1	2	9	10	3	6	5	4	7	8
F14	Ave	1.40 × 10^3^	1.40 × 10^3^	8.12 × 10^3^	4.26 × 10^3^	1.41 × 10^3^	2.63 × 10^3^	1.46 × 10^3^	1.43 × 10^3^	1.53 × 10^3^	1.98 × 10^3^
	Std	1.03 × 10^−1^	7.23 × 10^−1^	1.11 × 10^4^	2.20 × 10^3^	1.82 × 10^0^	1.65 × 10^3^	3.58 × 10^1^	1.10 × 10^1^	2.99 × 10^1^	6.48 × 10^2^
	Rank	1	2	10	9	3	8	5	4	6	7
F15	Ave	1.50 × 10^3^	1.50 × 10^3^	1.03 × 10^4^	8.90 × 10^3^	1.50 × 10^3^	3.18 × 10^3^	1.57 × 10^3^	1.55 × 10^3^	2.04 × 10^3^	5.38 × 10^3^
	Std	4.70 × 10^−2^	2.08 × 10^−1^	4.77 × 10^3^	5.49 × 10^3^	2.71 × 10^−1^	1.66 × 10^3^	4.52 × 10^1^	3.08 × 10^1^	3.62 × 10^2^	4.70 × 10^3^
	Rank	1	2	10	9	3	7	5	4	6	8
F16	Ave	1.60 × 10^3^	1.60 × 10^3^	2.04 × 10^3^	2.09 × 10^3^	1.60 × 10^3^	1.72 × 10^3^	1.83 × 10^3^	1.64 × 10^3^	1.73 × 10^3^	1.75 × 10^3^
	Std	1.11 × 10^−1^	3.35 × 10^−1^	1.11 × 10^2^	1.29 × 10^2^	3.50 × 10^−1^	1.19 × 10^2^	1.20 × 10^2^	6.58 × 10^1^	1.24 × 10^2^	6.74 × 10^1^
	Rank	1	2	9	10	3	5	8	4	6	7
F17	Ave	1.70 × 10^3^	1.70 × 10^3^	1.86 × 10^3^	1.82 × 10^3^	1.71 × 10^3^	1.74 × 10^3^	1.75 × 10^3^	1.74 × 10^3^	1.76 × 10^3^	1.86 × 10^3^
	Std	3.84 × 10^−1^	1.65 × 10^0^	4.44 × 10^1^	2.78 × 10^1^	3.25 × 10^0^	1.90 × 10^1^	2.26 × 10^1^	1.22 × 10^1^	3.39 × 10^1^	7.27 × 10^1^
	Rank	1	2	10	8	3	5	6	4	7	9
F18	Ave	1.80 × 10^3^	1.80 × 10^3^	4.46 × 10^6^	1.53 × 10^7^	1.80 × 10^3^	2.64 × 10^4^	4.97 × 10^3^	4.05 × 10^3^	2.02 × 10^4^	5.60 × 10^4^
	Std	1.09 × 10^−1^	5.46 × 10^−1^	6.83 × 10^6^	3.52 × 10^7^	1.12 × 10^0^	1.63 × 10^4^	4.20 × 10^3^	1.92 × 10^3^	1.66 × 10^4^	1.81 × 10^4^
	Rank	1	2	9	10	3	7	5	4	6	8
F19	Ave	1.90 × 10^3^	1.90 × 10^3^	2.85 × 10^4^	4.99 × 10^5^	1.90 × 10^3^	6.71 × 10^3^	2.19 × 10^3^	1.94 × 10^3^	2.53 × 10^3^	2.61 × 10^4^
	Std	3.39 × 10^−2^	9.22 × 10^−2^	3.37 × 10^4^	5.34 × 10^5^	2.28 × 10^−1^	5.57 × 10^3^	6.10 × 10^2^	2.65 × 10^1^	1.16 × 10^3^	1.40 × 10^4^
	Rank	1	2	9	10	3	7	5	4	6	8
F20	Ave	2.00 × 10^3^	2.00 × 10^3^	2.18 × 10^3^	2.22 × 10^3^	2.00 × 10^3^	2.05 × 10^3^	2.07 × 10^3^	2.03 × 10^3^	2.10 × 10^3^	2.19 × 10^3^
	Std	2.20 × 10^−12^	2.30 × 10^−1^	6.39 × 10^1^	3.92 × 10^1^	1.30 × 10^−1^	3.26 × 10^1^	6.49 × 10^1^	1.34 × 10^1^	6.33 × 10^1^	6.47 × 10^1^
	Rank	1	2	8	10	3	5	6	4	7	9
F21	Ave	2.20 × 10^3^	2.20 × 10^3^	2.35 × 10^3^	2.29 × 10^3^	2.21 × 10^3^	2.30 × 10^3^	2.29 × 10^3^	2.25 × 10^3^	2.26 × 10^3^	2.34 × 10^3^
	Std	5.94 × 10^−9^	1.38 × 10^−2^	3.05 × 10^1^	5.86 × 10^1^	2.49 × 10^1^	4.07 × 10^1^	5.33 × 10^1^	5.36 × 10^1^	6.20 × 10^1^	4.49 × 10^1^
	Rank	1	2	10	7	3	8	6	4	5	9
F22	Ave	2.25 × 10^3^	2.21 × 10^3^	2.61 × 10^3^	2.87 × 10^3^	2.30 × 10^3^	2.35 × 10^3^	2.30 × 10^3^	2.30 × 10^3^	2.30 × 10^3^	2.50 × 10^3^
	Std	5.13 × 10^1^	2.39 × 10^1^	1.68 × 10^2^	2.02 × 10^2^	5.58 × 10^−1^	1.81 × 10^2^	2.02 × 10^1^	1.12 × 10^0^	1.83 × 10^1^	9.39 × 10^1^
	Rank	2	1	9	10	4	7	3	6	5	8
F23	Ave	2.60 × 10^3^	2.61 × 10^3^	2.69 × 10^3^	2.69 × 10^3^	2.61 × 10^3^	2.62 × 10^3^	2.62 × 10^3^	2.62 × 10^3^	2.62 × 10^3^	2.64 × 10^3^
	Std	1.08 × 10^0^	1.13 × 10^0^	2.25 × 10^1^	9.98 × 10^0^	3.18 × 10^0^	8.09 × 10^0^	1.31 × 10^1^	6.32 × 10^0^	6.65 × 10^0^	6.20 × 10^0^
	Rank	1	2	9	10	3	4	6	5	7	8
F24	Ave	2.50 × 10^3^	2.50 × 10^3^	2.82 × 10^3^	2.85 × 10^3^	2.62 × 10^3^	2.74 × 10^3^	2.72 × 10^3^	2.73 × 10^3^	2.72 × 10^3^	2.78 × 10^3^
	Std	9.21 × 10^−13^	3.92 × 10^−8^	3.07 × 10^1^	5.95 × 10^1^	1.22 × 10^2^	1.11 × 10^1^	7.72 × 10^1^	5.34 × 10^1^	9.35 × 10^1^	6.29 × 10^0^
	Rank	1	2	9	10	3	7	5	6	4	8
F25	Ave	2.90 × 10^3^	2.88 × 10^3^	3.11 × 10^3^	3.30 × 10^3^	2.90 × 10^3^	2.93 × 10^3^	2.92 × 10^3^	2.92 × 10^3^	2.92 × 10^3^	3.04 × 10^3^
	Std	9.33 × 10^−13^	6.65 × 10^1^	1.04 × 10^2^	1.13 × 10^2^	1.66 × 10^1^	1.60 × 10^1^	3.27 × 10^1^	2.39 × 10^1^	2.47 × 10^1^	3.27 × 10^1^
	Rank	2	1	9	10	3	7	4	6	5	8
F26	Ave	2.81 × 10^3^	2.83 × 10^3^	3.66 × 10^3^	4.01 × 10^3^	2.90 × 10^3^	3.02 × 10^3^	2.88 × 10^3^	2.99 × 10^3^	2.96 × 10^3^	3.29 × 10^3^
	Std	1.30 × 10^2^	1.08 × 10^2^	2.85 × 10^2^	2.96 × 10^2^	2.24 × 10^1^	3.03 × 10^2^	7.36 × 10^1^	7.85 × 10^1^	2.28 × 10^2^	4.75 × 10^2^
	Rank	1	2	9	10	4	7	3	6	5	8
F27	Ave	3.09 × 10^3^	3.09 × 10^3^	3.16 × 10^3^	3.19 × 10^3^	3.09 × 10^3^	3.09 × 10^3^	3.10 × 10^3^	3.10 × 10^3^	3.10 × 10^3^	3.10 × 10^3^
	Std	9.42 × 10^−1^	8.96 × 10^−1^	2.43 × 10^1^	5.12 × 10^1^	1.51 × 10^0^	3.54 × 10^0^	1.67 × 10^1^	1.20 × 10^1^	1.52 × 10^1^	1.10 × 10^1^
	Rank	1	2	9	10	3	4	8	6	5	7
F28	Ave	3.10 × 10^3^	3.10 × 10^3^	3.53 × 10^3^	3.73 × 10^3^	3.10 × 10^3^	3.33 × 10^3^	3.22 × 10^3^	3.20 × 10^3^	3.30 × 10^3^	3.33 × 10^3^
	Std	9.46 × 10^−12^	5.12 × 10^−5^	1.13 × 10^2^	9.77 × 10^1^	7.76 × 10^−9^	1.00 × 10^2^	1.41 × 10^2^	1.00 × 10^2^	1.75 × 10^2^	7.95 × 10^1^
	Rank	1	3	9	10	2	8	5	4	6	7
F29	Ave	3.14 × 10^3^	3.15 × 10^3^	3.39 × 10^3^	3.33 × 10^3^	3.17 × 10^3^	3.18 × 10^3^	3.21 × 10^3^	3.17 × 10^3^	3.21 × 10^3^	3.25 × 10^3^
	Std	1.75 × 10^0^	7.83 × 10^0^	7.79 × 10^1^	7.80 × 10^1^	6.72 × 10^0^	3.31 × 10^1^	5.19 × 10^1^	1.92 × 10^1^	4.20 × 10^1^	6.61 × 10^1^
	Rank	1	2	10	9	3	5	6	4	7	8
F30	Ave	3.41 × 10^3^	3.51 × 10^3^	1.35 × 10^7^	3.61 × 10^6^	3.69 × 10^3^	4.29 × 10^5^	2.96 × 10^5^	1.00 × 10^5^	2.73 × 10^5^	4.49 × 10^5^
	Std	3.52 × 10^0^	5.74 × 10^1^	1.27 × 10^7^	4.38 × 10^6^	1.57 × 10^2^	7.89 × 10^5^	4.63 × 10^5^	2.25 × 10^5^	4.75 × 10^5^	4.78 × 10^5^
	Rank	1	2	10	9	3	7	6	4	5	8
Mean	Ranking	1.1071	2.0714	9.1429	9.3571	3.1786	6.0000	4.9643	4.6429	6.2143	8.3214
Final	Rank	1	2	9	10	3	6	5	4	7	8

**Table 4:** Results of the CEC-2020 test suite.

Fun	Metrics	RLNOA	NOA	SO	RSA	CPO	GWO	PSO	RLTLBO	RLMPSO	RLCGWO
F1	Ave	1.00 × 10^2^	1.44 × 10^2^	1.70 × 10^10^	2.91 × 10^10^	1.04 × 10^2^	1.40 × 10^8^	3.58 × 10^3^	3.41 × 10^3^	6.56 × 10^3^	1.57 × 10^10^
	Std	2.14 × 10^−1^	3.57 × 10^1^	5.57 × 10^9^	4.45 × 10^9^	3.33 × 10^0^	3.75 × 10^8^	3.13 × 10^3^	3.52 × 10^3^	4.52 × 10^3^	2.63 × 10^9^
	Rank	1	3	9	10	2	7	5	4	6	8
F2	Ave	1.36 × 10^3^	1.96 × 10^3^	5.71 × 10^3^	5.53 × 10^3^	2.70 × 10^3^	2.54 × 10^3^	2.45 × 10^3^	2.61 × 10^3^	3.49 × 10^3^	5.53 × 10^3^
	Std	1.14 × 10^2^	1.62 × 10^2^	3.81 × 10^2^	2.06 × 10^2^	2.39 × 10^2^	5.79 × 10^2^	3.68 × 10^2^	5.94 × 10^2^	6.67 × 10^2^	4.30 × 10^2^
	Rank	1	2	10	9	6	4	3	5	7	8
F3	Ave	7.31 × 10^2^	7.42 × 10^2^	1.04 × 10^3^	1.01 × 10^3^	7.63 × 10^2^	7.67 × 10^2^	7.53 × 10^2^	7.99 × 10^2^	8.26 × 10^2^	1.74 × 10^3^
	Std	3.04 × 10^0^	1.01 × 10^1^	3.51 × 10^1^	2.94 × 10^1^	4.60 × 10^0^	2.22 × 10^1^	1.03 × 10^1^	3.05 × 10^1^	3.73 × 10^1^	1.90 × 10^2^
	Rank	1	2	9	8	4	5	3	6	7	10
F4	Ave	1.90 × 10^3^	1.90 × 10^3^	1.54 × 10^5^	3.54 × 10^5^	1.91 × 10^3^	1.91 × 10^3^	1.90 × 10^3^	1.92 × 10^3^	1.91 × 10^3^	3.63 × 10^4^
	Std	3.02 × 10^−1^	4.32 × 10^−1^	1.13 × 10^5^	1.81 × 10^5^	6.18 × 10^−1^	3.06 × 10^0^	6.75 × 10^−1^	1.04 × 10^1^	3.31 × 10^0^	2.18 × 10^4^
	Rank	2	3	9	10	4	5	1	7	6	8
F5	Ave	2.12 × 10^3^	2.40 × 10^3^	3.16 × 10^6^	5.01 × 10^6^	3.02 × 10^3^	3.95 × 10^5^	8.93 × 10^4^	6.03 × 10^4^	2.74 × 10^5^	2.75 × 10^6^
	Std	5.42 × 10^1^	1.28 × 10^2^	1.41 × 10^6^	2.14 × 10^6^	2.16 × 10^2^	6.93 × 10^5^	5.25 × 10^4^	3.58 × 10^4^	1.55 × 10^5^	2.52 × 10^6^
	Rank	1	2	9	10	3	7	5	4	6	8
F6	Ave	1.60 × 10^3^	1.60 × 10^3^	2.88 × 10^3^	3.12 × 10^3^	1.61 × 10^3^	1.86 × 10^3^	1.88 × 10^3^	1.75 × 10^3^	1.94 × 10^3^	2.38 × 10^3^
	Std	1.82 × 10^−1^	4.85 × 10^−1^	3.61 × 10^2^	4.69 × 10^2^	4.30 × 10^0^	1.49 × 10^2^	1.58 × 10^2^	1.10 × 10^2^	1.58 × 10^2^	2.12 × 10^2^
	Rank	1	2	9	10	3	5	6	4	7	8
F7	Ave	2.25 × 10^3^	2.39 × 10^3^	1.33 × 10^6^	3.21 × 10^6^	2.69 × 10^3^	1.36 × 10^5^	5.24 × 10^4^	1.85 × 10^4^	9.30 × 10^4^	1.04 × 10^6^
	Std	3.46 × 10^1^	8.91 × 10^1^	1.32 × 10^6^	3.63 × 10^6^	1.06 × 10^2^	8.51 × 10^4^	7.68 × 10^4^	1.91 × 10^4^	7.48 × 10^4^	6.58 × 10^5^
	Rank	1	2	9	10	3	7	5	4	6	8
F8	Ave	2.30 × 10^3^	2.30 × 10^3^	4.78 × 10^3^	5.30 × 10^3^	2.30 × 10^3^	2.71 × 10^3^	2.40 × 10^3^	2.30 × 10^3^	2.30 × 10^3^	5.47 × 10^3^
	Std	1.43 × 10^1^	1.44 × 10^−4^	9.68 × 10^2^	7.07 × 10^2^	4.04 × 10^−6^	7.46 × 10^2^	4.47 × 10^2^	5.71 × 10^0^	1.23 × 10^0^	1.08 × 10^3^
	Rank	1	3	8	9	2	7	6	5	4	10
F9	Ave	2.81 × 10^3^	2.81 × 10^3^	3.13 × 10^3^	3.19 × 10^3^	2.86 × 10^3^	2.85 × 10^3^	2.86 × 10^3^	2.86 × 10^3^	2.88 × 10^3^	2.94 × 10^3^
	Std	4.14 × 10^0^	6.80 × 10^1^	6.79 × 10^1^	1.85 × 10^2^	1.01 × 10^1^	3.68 × 10^1^	3.03 × 10^1^	1.97 × 10^1^	3.45 × 10^1^	1.33 × 10^1^
	Rank	2	1	9	10	6	3	4	5	7	8
F10	Ave	2.91 × 10^3^	2.91 × 10^3^	4.26 × 10^3^	4.82 × 10^3^	2.93 × 10^3^	2.95 × 10^3^	2.93 × 10^3^	2.98 × 10^3^	2.95 × 10^3^	4.15 × 10^3^
	Std	4.49 × 10^0^	3.87 × 10^−2^	5.13 × 10^2^	7.40 × 10^2^	2.54 × 10^1^	3.01 × 10^1^	3.04 × 10^1^	3.81 × 10^1^	3.41 × 10^1^	4.77 × 10^2^
	Rank	1	2	9	10	4	5	3	7	6	8
Mean	Ranking	1.2222	2.2222	9.0000	9.5556	3.6667	5.5556	4.2222	4.8889	6.2222	8.4444
Final	Rank	1	2	9	10	3	6	4	5	7	8

**Table 5:** Results of the RLNOA with different values for the parameter $P_{rp}$.

$P_{rp}$	Fun			
	F1	F4	F17	F23
0.8	1.00 × 10^2^	4.00 × 10^2^	1.72 × 10^3^	2.50 × 10^3^
0.6	1.00 × 10^2^	4.00 × 10^2^	1.72 × 10^3^	2.50 × 10^3^
0.4	1.00 × 10^2^	4.00 × 10^2^	1.72 × 10^3^	2.50 × 10^3^
0.2	1.00 × 10^2^	4.00 × 10^2^	1.71 × 10^3^	2.50 × 10^3^

**Table 6:** Results of the RLNOA with different values for the parameter δ.

δ	Fun			
	F1	F4	F17	F23
0.5	1.00 × 10^2^	4.00 × 10^2^	1.71 × 10^3^	2.50 × 10^3^
0.2	1.00 × 10^2^	4.00 × 10^2^	1.71 × 10^3^	2.50 × 10^3^
0.1	1.00 × 10^2^	4.00 × 10^2^	1.71 × 10^3^	2.50 × 10^3^
0.05	1.00 × 10^2^	4.00 × 10^2^	1.71 × 10^3^	2.50 × 10^3^

**Table 7:** Results of the RLNOA with different values for the parameter k.

k	Fun			
	F1	F4	F17	F23
5	1.00 × 10^2^	4.00 × 10^2^	1.71 × 10^3^	2.50 × 10^3^
10	1.00 × 10^2^	4.00 × 10^2^	1.71 × 10^3^	2.50 × 10^3^
20	1.00 × 10^2^	4.00 × 10^2^	1.71 × 10^3^	2.50 × 10^3^
50	1.00 × 10^2^	4.00 × 10^2^	1.71 × 10^3^	2.50 × 10^3^

**Table 8:** Results of the RLNOA with different values for the parameter ζ.

ζ	Fun			
	F1	F4	F17	F23
1	1.00 × 10^2^	4.00 × 10^2^	1.71 × 10^3^	2.50 × 10^3^
2	1.01 × 10^2^	4.00 × 10^2^	1.72 × 10^3^	2.50 × 10^3^
4	1.04 × 10^2^	4.00 × 10^2^	1.72 × 10^3^	2.50 × 10^3^
8	1.17 × 10^2^	4.00 × 10^2^	1.72 × 10^3^	2.50 × 10^3^

## Data Availability

The data presented in this study are available on request from the corresponding author.

## Appendix A

**Table A1:** The nomenclature of this study.

***Indices***		*A*, *B*, and *C*	Integers randomly selected in the range of [0, *NP*]
i	Index of individuals in the population	$L_{j}$	The lower bound of the optimization problem in the *j*th dimension
j	Index of dimensions	*NP*	The population size
l	Index of individuals in the neighborhood set	$P_{a2}$	A probability value
t	Index of iteration	$P_{rp}$	A global exploration threshold
***Sets***		$T_{\max}$	The maximum iterations
${\left\{x_{i}^{t}\right\}}_{i=1}^{NP}$	The population in the iteration t	$U_{j}$	The upper bound of the optimization problem in the *j*th dimension
${\left\{x_{l}^{t}\right\}}_{l=1}^{k}$	The neighborhood set of $x_{i}^{t}$	***Variables***	
***Parameters***		$a_{t}$ and $a_{t+1}$	The current and next actions respectively
α	The learning rate	$fit_{i}^{t}$	The relative changes of fitness value
γ	The discount factor	$ld_{i}^{t}$	The relative changes of local diversity
δ and ζ	Control parameter	$s_{t}$ and $s_{t+1}$	The current and next states respectively
ε, λ, and $\tau_{3}$	A parameter generated by the levy flight	$x_{i,j}^{t+1\left(FSnew_{1}\right)}$	The new position of the *i*th individual generated in the foraging phase
θ	A parameter chosen in the range of [0, π]	$x_{i,j}^{t}$	The *j*th dimension of the *i*th individual in the iteration t
ξ and $P_{a1}$	A parameter that linearly decreased from 1 to 0	$x_{i}^{t+1\left(FSnew_{2}\right)}$	The new position of the *i*th individual generated in the storage phase
$\tau_{1}$	A random number selected within the range [0, 1]	$x_{m,j}^{t}$	The mean position of the *j*th dimensions for current population in the iteration t
$\tau_{2}$	A random number generated based on a normal distribution	$D_{i}^{t}$	The local diversity of individual $x_{i}^{t}$
d	The dimension of the search space	$N_{exploration}$	The size of the exploration sub-population
k	The number of near neighbors	$Q\left(\cdot \right)$	The corresponding value in the Q-table
rand and r	Random numbers selected within the range [0, 1]	$R_{t+1}$	The reward acquired after performing action $a_{t}$
$randi_{1↔2}$	An integer chosen randomly between zero and one		
